# Machine learning models for diagnosis and risk prediction in eating disorders, depression, and alcohol use disorder

**DOI:** 10.1016/j.jad.2024.12.053

**Published:** 2024-12-17

**Authors:** Zuo Zhang, Lauren Robinson, Robert Whelan, Lee Jollans, Zijian Wang, Frauke Nees, Congying Chu, Marina Bobou, Dongping Du, Ilinca Cristea, Tobias Banaschewski, Gareth J. Barker, Arun L.W. Bokde, Antoine Grigis, Hugh Garavan, Andreas Heinz, Rüdiger Brühl, Jean-Luc Martinot, Marie-Laure Paillère Martinot, Eric Artiges, Dimitri Papadopoulos Orfanos, Luise Poustka, Sarah Hohmann, Sabina Millenet, Juliane H. Fröhner, Michael N. Smolka, Nilakshi Vaidya, Henrik Walter, Jeanne Winterer, M. John Broulidakis, Betteke Maria van Noort, Argyris Stringaris, Jani Penttilä, Yvonne Grimmer, Corinna Insensee, Andreas Becker, Yuning Zhang, Sinead King, Julia Sinclair, Gunter Schumann, Ulrike Schmidt, Sylvane Desrivières

**Affiliations:** a Social, Genetic and Developmental Psychiatry Centre, Institute of Psychiatry, Psychology & Neuroscience, King’s College London, De Crespigny Park, London, SE5 8AF, UK; b School of Psychology, Institute for Mental Health, University of Birmingham, Birmingham, UK; c Department of Psychological Medicine, Centre for Research in Eating and Weight Disorders, Institute of Psychiatry, Psychology & Neuroscience, King’s College London, UK; d South London and Maudsley NHS Foundation Trust, London, UK; e Oxford Institute of Clinical Psychology Training and Research, Oxford University, Oxford, UK; f School of Psychology and Global Brain Health Institute, Trinity College Dublin, Ireland; g School of Computer Science and Technology, Donghua University, Shanghai, China; h Department of Child and Adolescent Psychiatry and Psychotherapy, Central Institute of Mental Health, Medical Faculty Mannheim, Heidelberg University, Square J5, 68159 Mannheim, Germany; i Institute of Cognitive and Clinical Neuroscience, Central Institute of Mental Health, Medical Faculty Mannheim, Heidelberg University, Square J5, Mannheim, Germany; j Institute of Medical Psychology and Medical Sociology, University Medical Center Schleswig-Holstein, Kiel University, Kiel, Germany; k University of Chinese Academy of Sciences, 100190 Beijing, China; l Brainnetome Center, Institute of Automation, Chinese Academy of Sciences, 100190 Beijing, China; m National Laboratory of Pattern Recognition, Institute of Automation, Chinese Academy of Sciences, 100190 Beijing, China; n Research Department of Clinical, Educational and Health Psychology, University College London, London, United Kingdom; o Department of Electrical and Computer Engineering, Virginia Polytechnic Institute and State University, Arlington, VA 22203, USA; p Department of Neuroimaging, Institute of Psychiatry, Psychology & Neuroscience, King’s College London, London, UK; q Discipline of Psychiatry, School of Medicine and Trinity College Institute of Neuroscience, Trinity College Dublin, Dublin, Ireland; r NeuroSpin, CEA, Université Paris-Saclay, F-91191 Gif-sur-Yvette, France; s Departments of Psychiatry and Psychology, University of Vermont, 05405 Burlington, Vermont, USA; t Charité – Universitätsmedizin Berlin, corporate member of Freie Universität Berlin, Humboldt-Universität zu Berlin, and Berlin Institute of Health, Department of Psychiatry and Psychotherapy, Campus Charité Mitte, Charitéplatz 1, Berlin, Germany; u Physikalisch-Technische Bundesanstalt (PTB), Braunschweig and Berlin, Germany; v Institut National de la Santé et de la Recherche Médicale, INSERM U1299 “Developmental trajectories & psychiatry”; Université Paris-Saclay, Université Paris Cité, Ecole Normale supérieure Paris-Saclay, CNRS; Centre Borelli UMR9010; Gif-sur-Yvette, France; w AP-HP. Sorbonne Université, Department of Child and Adolescent Psychiatry, Pitié-Salpêtrière Hospital, Paris, France; x Psychiatry Department, EPS Barthélemy Durand, Etampes, France; y Department of Child and Adolescent Psychiatry and Psychotherapy, University Medical Centre Göttingen, von-Siebold-Str. 5, 37075, Göttingen, Germany; z Department of Psychiatry and Neuroimaging Center, Technische Universität Dresden, Dresden, Germany; aa Centre for Population Neuroscience and Stratified Medicine (PONS), Department of Psychiatry and Neuroscience, Charité Universitätsmedizin Berlin, Germany; ab Department of Education and Psychology, Freie Universität Berlin, Berlin, Germany; ac Clinical and Experimental Sciences, Faculty of Medicine, University of Southampton, Southampton, UK; ad Department of Psychology, College of Science, Northeastern University, Boston, MA, USA; ae Department of Psychology, MSB Medical School Berlin, Rüdesheimer Str. 50, 14197 Berlin, Germany; af Division of Psychiatry and Department of Clinical, Educational & Health Psychology, University College London, UK; ag Department of Social and Health Care, Psychosocial Services Adolescent Outpatient Clinic Kauppakatu 14, Lahti, Finland; ah Psychology Department, B44 University Rd, University of Southampton, Southampton SO17 1PS, UK; ai School of Medicine, Centre for Neuroimaging, Cognition and Genomics, National University of Ireland (NUI) Galway; aj Beaumont Hospital, Royal College of Surgeons, Ireland; ak Centre for Population Neuroscience and Precision Medicine (PONS), Institute for Science and Technology of Brain-inspired Intelligence (ISTBI), Fudan University, Shanghai, China

**Keywords:** eating disorders, major depressive disorder, alcohol use disorder, risk factors, predictive modeling.

## Abstract

**Background:**

Early diagnosis and treatment of mental illnesses is hampered by the lack of reliable markers. This study used machine learning models to uncover diagnostic and risk prediction markers for eating disorders (EDs), major depressive disorder (MDD), and alcohol use disorder (AUD).

**Methods:**

Case-control samples (aged 18-25 years), including participants with Anorexia Nervosa (AN), Bulimia Nervosa (BN), MDD, AUD, and matched controls, were used for diagnostic classification. For risk prediction, we used a longitudinal population-based sample (IMAGEN study), assessing adolescents at ages 14, 16 and 19. Regularized logistic regression models incorporated broad data domains spanning psychopathology, personality, cognition, substance use, and environment.

**Results:**

The classification of EDs was highly accurate, even when excluding body mass index from the analysis. The area under the receiver operating characteristic curves (AUC-ROC [95% CI]) reached 0.92 [0.86-0.97] for AN and 0.91 [0.85-0.96] for BN. The classification accuracies for MDD (0.91 [0.88-0.94]) and AUD (0.80 [0.74-0.85]) were also high. The models demonstrated high transdiagnostic potential, as those trained for EDs were also accurate in classifying AUD and MDD from healthy controls, and vice versa (AUC-ROCs, 0.75-0.93). Shared predictors, such as neuroticism, hopelessness, and symptoms of attention-deficit/hyperactivity disorder, were identified as reliable classifiers. In the longitudinal population sample, the models exhibited moderate performance in predicting the development of future ED symptoms (0.71 [0.67-0.75]), depressive symptoms (0.64 [0.60-0.68]), and harmful drinking (0.67 [0.64-0.70]).

**Conclusions:**

Our findings demonstrate the potential of combining multi-domain data for precise diagnostic and risk prediction applications in psychiatry.

## 1 Introduction

Eating disorders (EDs), including Anorexia Nervosa (AN), Bulimia Nervosa (BN), Binge-Eating Disorder (BED) and related sub-threshold syndromes, are a major healthcare challenge with significant public health and economic impacts. These complex and disabling disorders affect 6-18% of young women and up to 2% of young men by early adulthood (Galmiche et al., 2019). With a typical age of onset between 15 and 25 years, EDs seriously impact young people’s life chances, their families, and the wider society (Santomauro et al., 2021). Mortality rates in people with EDs are twice as high as in the general population, and about six times higher for people with AN (Arcelus et al., 2011). Psychiatric comorbidities such as anxiety, mood, and substance use disorders are common and negatively impact ED outcomes (Momen et al., 2022). This complexity makes EDs hard to detect and treat, and relapse occurs frequently (Khalsa et al., 2017). Early detection and more accurate patient classification are key priorities in the development of effective interventions.

A multifactorial neurodevelopmental model has been proposed to explain the complexity of EDs (Connan et al., 2003). Widely accepted risk factors include sex, body mass index (BMI), weight/shape concerns, low self-esteem, a history of depression, anxiety, attention-deficit/hyperactivity disorder (ADHD) symptoms, and disordered eating behaviors (McClelland et al., 2020). Personality traits, notably neuroticism, have also been implicated in EDs (Farstad et al., 2016). At the environmental level, traumatic experiences of neglect and abuse in childhood are linked to higher risks of ED pathology (Caslini et al., 2016; Pignatelli et al., 2017). However, while there is evidence for multiple biopsychosocial risk factors, most studies typically focus on only a single or a small number of risk factors. It is still unknown which combinations of factors will most accurately reflect ED susceptibility/risk or improve diagnostic classification, which is a focus of the current study.

Over half of individuals with EDs have a co-occurring psychiatric disorder, with anxiety and mood disorders being the most prevalent, both affecting over 50% of individuals with EDs (Hambleton et al., 2022). Particularly, patients with EDs and depressive disorders exhibit similar levels of core depressive symptoms, including sadness and loss of pleasure (Voderholzer et al., 2019). This comorbidity may result from shared risk factors and underlying mechanisms, such as genetic predispositions (Thornton et al., 2016), exposure to environmental stressors such as adverse events and trauma, and malfunctions in reward sensitivity and emotional regulation (Donofry et al., 2016). There is also a reciprocal relationship between EDs and depressive disorders (Miskovic-Wheatley et al., 2023): patients with depression may engage in disordered eating as a coping strategy, while having a negative body image in ED patients is a main contributor to depressive symptoms (Junne et al., 2016). Alcohol use disorder (AUD) is also often comorbid, affecting about one in five individuals with EDs, particularly patients with the binge / purge ED subtype (Bahji et al., 2019). Shared mechanisms suggested to underlie this comorbidity include impulsivity and novelty-seeking, reward sensitivity, and deficits in executive function and emotion dysregulation (Claudat et al., 2023). Alcohol may be also used to reduce ED-related anxiety and affective symptoms (Devoe et al., 2021). These high comorbidities and shared mechanisms highlight the importance of a transdiagnostic approach for the treatment and prevention of these disorders. The current study aims to identify psychosocial correlates and early risk factors that are shared and specific across EDs, major depressive disorder (MDD), and AUD.

Machine learning methods and the emergence of large data cohorts have provided opportunities to build multivariate risk profiles for psychiatric disorders. In ED research, these methods have been used in cross-sectional classification and regression models derived from distinct datasets, such as questionnaires (Krug et al., 2021; Ren et al., 2022; Voica et al., 2021), social media (Kelley et al., 2022), or neuroimaging data (Cerasa et al., 2015; Cyr et al., 2018). Longitudinal models have also been built to predict illness course (Haynos et al., 2021) and treatment outcomes (Forrest et al., 2021). Yet, to our knowledge, no ED study to date has combined a wide range of data domains to build models for diagnostic classification or risk prediction.

We addressed this research gap by deriving machine-learning-based models from broad domains of psychosocial data, collected from two samples that underwent similar assessments: 1) a clinical sample comprising young people with AN, BN, MDD, and AUD from the ESTRA and STRATIFY studies, and 2) participants aged 14 to 19 years from the longitudinal population-based adolescent cohort IMAGEN. Analyses in the clinical sample were conducted with the aim of identifying multidomain markers for diagnostic prediction of EDs, MDD, and AUD, and describe their most important classifiers. Analyses in the longitudinal population sample aimed at identifying reliable markers for susceptibility/risk of developing symptoms of EDs, MDD, and AUD.

## 2 Methods

### 2.1 Participants

Participants were assessed as part of the ESTRA, STRATIFY, and IMAGEN studies. These were ‘sibling’ studies that were designed with shared assessments and protocols to enable comparability.

#### Case-control studies

Our clinical sample included participants with AN and BN, recruited as part of the ESTRA study. All the participants were female, aged 18-25 years, and recruited at the London study site. Healthy controls (HC) for the ED patients were selected from the IMAGEN study (see below) at the third follow-up (~23 years old) and were female, recruited in London, and screened negative for all psychiatric diagnoses based on the Mini International Neuropsychiatric Interview (MINI) (Sheehan et al., 1998). Participants with MDD and AUD, and their corresponding HCs were aged 18-25 years and recruited as part of the STRATIFY study from three study sites: London, Southampton, UK and Berlin, Germany. HCs for MDD and AUD were recruited based on the following inclusion criteria: a) a total score < 5 on the Patient Health Questionnaire-9 (PHQ-9) (Kroenke et al., 2001); b) a total score < 5 on the Alcohol Use Disorders Identification Test (AUDIT) (Babor et al., 2001); c) no self-reported current or past mental health issues; d) having no first or second order family members with mental health issues; e) no learning difficulties; f) no regular medication for serious physical health issues; and g) no regular recreational drug use (see Supplementary Methods for more details).

#### Longitudinal cohort study

This population sample was derived from IMAGEN, a longitudinal neuroimaging and genetics study of adolescents recruited from eight study sites in Europe (Schumann et al., 2010). The data used in the longitudinal prediction analysis were acquired at ages 14, 16, and 19 years. Eating disorder symptoms were assessed by self-report of concerns over one’s shape, weight, and eating, and disordered eating behaviors (binge eating, purging, and dieting) based on the Development and Wellbeing Assessment (DAWBA) (Goodman et al., 2000). ‘Developers’ were defined as individuals who did not report any ED symptom at age 14, but reported one or more symptoms at age 16 or 19. They were compared to controls, who remained asymptomatic across the three ages. Depressive symptoms and harmful drinking were measured using DAWBA bands for depression (Goodman et al., 2011) and the Alcohol Use Disorders Identification Test (AUDIT) (Babor et al., 2001), respectively. Developers of depression and harmful drinking were defined as individuals scoring low on depressive symptoms and harmful drinking at age 14 respectively, but high at age 16/19. Controls for these groups scored low on depressive symptoms and harmful drinking, respectively, across the three ages (for more details, see Supplementary Methods). Data collected at age 14 were used to predict whether participants developed each mental health symptom at age 16/19.

### 2.2 Measures

Demographic information, including sex assigned at birth, age, and ethnicity was acquired from self-report. Our analyses combined a wide range of data domains comprising cognition, environment, personality, psychopathology, substance use, and BMI (for full details, see Supplementary Methods). Full lists of variables and percentages of missing data are provided in Supplementary Tables 3-5.

### 2.3 Ethical approval

The authors assert that all procedures contributing to this work comply with the ethical standards of the relevant national and institutional committees on human experimentation and with the Helsinki Declaration of 1975, as revised in 2008. All procedures involving human subjects were approved for the ESTRA study by the North West-Greater Manchester South Research Ethics Committee (reference number: 23/NW/0232) in the UK. The STRATIFY study was approved by the London Westminster Research Ethics Committee (reference: 17/LO/0552) in the UK and Charité Ethikkommission (reference: EA1/030/18) in Germany. The IMAGEN study was approved by the local research ethics committee at each study site (London, Nottingham, UK; Dublin, Ireland; Paris, France; Berlin, Hamburg, Mannheim, and Dresden, Germany). Written informed consent was obtained from all participants aged 18 years and above. For all participants under 18 years, written assent was obtained from them and written consent from their parents/guardians.

### 2.4 Data Analysis

A logistic regression model with L1 and L2 regularization, namely Elastic Net was used, and implemented in the glmnet (version 4.1-7) package (Friedman et al., 2010) in R (version 4.2.1). Model performance was assessed by area under the receiver operating characteristic curve (AUC-ROC) and area under the precision and recall curve (AUC-PR). These performance metrics were derived from a nested cross-validation (CV) procedure. The whole dataset was randomly split into 10 equally sized subsets, while keeping the same ratio between cases and controls across subsets. One subset was reserved for model testing, and the remaining data was used for model training.

The data preparation procedure included imputation of missing values, partialling out the effects of confounding variables, standardization, and handling extreme values. These procedures were conducted on the training and testing data separately, to ensure that no information from the testing data was exploited in the training phase. First, missing data were imputed in the training and testing data separately, by using a Random Forest-based method implemented in the missForest package (Stekhoven and Bühlmann, 2012) in R (version 4.2.1). Second, the effects of confounding variables were partialled out from the training and testing data separately by using linear regression, following the procedure recommended by Snoek, Miletić, & Scholte (2019), with details provided in the Supplementary Methods. Third, each feature in the training data was standardized into z-scores. The mean and standard deviation of each feature in the training data were used to standardize the testing data. Last, to mitigate the impact of extreme values on model fitting, the z-scores smaller than -3 or larger than 3 were recoded as -3 and 3, respectively.

A five-fold inner CV was nested within the training data to select the optimal hyperparameters (L1 and L2 regularization terms) for the Elastic Net model, with the goal of maximizing AUC-ROC on the training data. By using the optimal hyper-parameters, an Elastic Net model was fitted on all the training data (90% of the whole dataset). The model’s performance was assessed with the remaining subset (10% of the whole dataset). This process was repeated until each subset had been used as the testing data. In the case where the model involved a single predictor of BMI, an ordinary logistic regression model was used instead. The same 10-fold CV procedure was employed as above, but the nested CV and hyperparameter tuning procedures were omitted.

The above CV procedure was repeated 10 times to mitigate the effect of data splitting. The model’s performance metrics were averaged across the 10 repetitions. Confidence intervals were obtained from 2000 bootstraps, and *p*-values from permutation tests with 5000 permutes. More details are provided in Supplementary Methods.

#### Classification of ED patients

**Figure 1:**
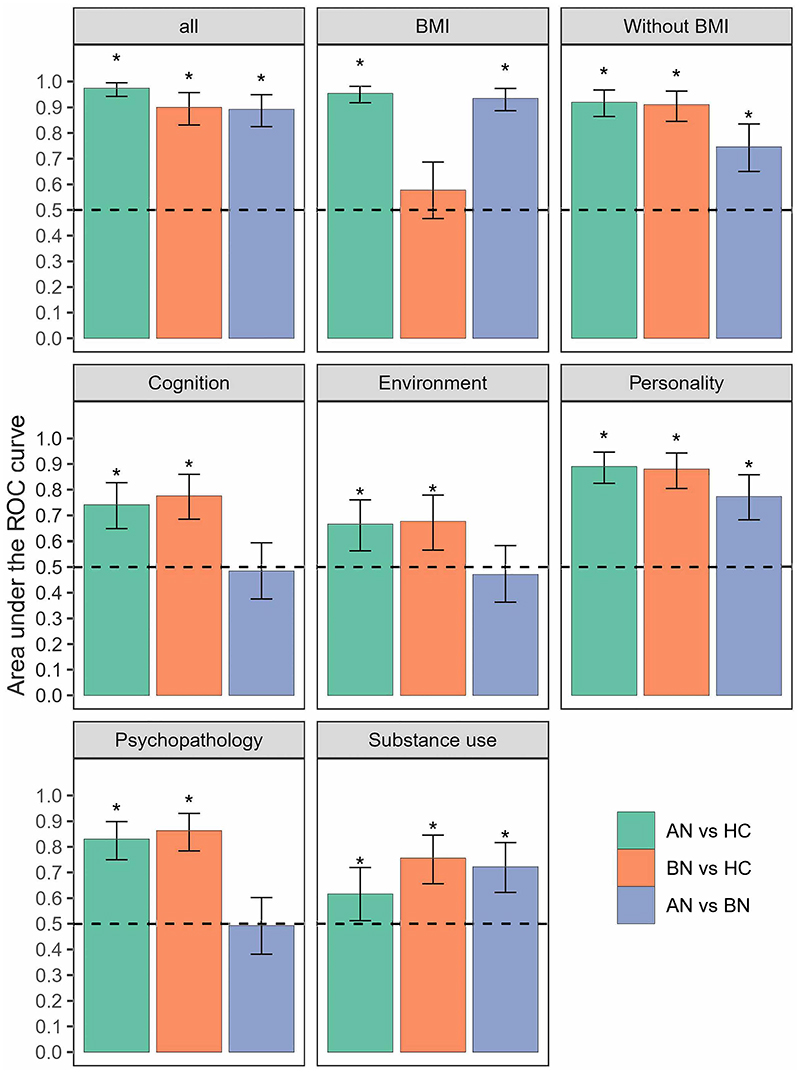
Classification performance on the AN, BN, and HC samples. Asterisks (*) indicate the performance is significantly above chance after correction with a false discovery rate (FDR) <0.05 for the 24 tests. Error bars indicate 95% bootstrap confidence intervals. Dashed lines indicate chance level performance (0.5). ROC curve, receiver operating characteristic curve. AN, anorexia nervosa. BN, bulimia nervosa. HC, healthy controls.

Firstly, we included all the variables (N=47) in building the classification model and considered age as a confounding variable. Given BMI is a diagnostic criterion for AN, a second model was run after excluding BMI. We further built models that involved each data domain alone to test if they could distinguish ED groups. A total of 18 models were built (Figure 1). A variable was identified as a reliable contributor to the Elastic Net model if it had a non-zero coefficient in at least 90% of all the CV folds (Whelan et al., 2014). The model’s coefficient for each feature was averaged across all the CV folds to obtain the median value, representing the feature’s importance.

#### Classification of MDD and AUD patients

We excluded 14 variables with excessive missing data, such as measures of cognitive performance and traumatic experiences (as indicated in Supplementary Table 4). To avoid circular analysis, we excluded depressive and emotional symptoms from the MDD vs. HC analysis, and excluded the harmful drinking scale from the AUD vs. HC analysis. This resulted in a set of 35 predictors for the MDD vs. HC analysis and 36 for the AUD vs. HC analysis. Due to unbalanced sex in the MDD and HC groups (75 % vs. 59% females, Supplementary Table 1), sex was considered as a confounding variable, in addition to age and study site.

#### Transdiagnostic models

We tested whether the model derived from the AN vs. HC and BN vs. HC analyses could also distinguish MDD and AUD from HC, and vice versa. Given that low BMI is a diagnostic criterion of AN but is unrelated to MDD and AUD, BMI was excluded from the transdiagnostic analysis. To avoid circular analysis, depressive and emotional symptoms were removed from the MDD vs. HC analysis, and the harmful drinking scale was removed from the AUD vs. HC analysis. In addition, variables with excessive missing data in the AUD and MDD samples (Supplementary Table 4) were excluded. A model for AN vs. HC and BN vs. HC classification was constructed, respectively, by using the whole dataset and the median values of the optimal hyperparameters obtained from the CV folds. We tested whether these models could distinguish MDD and AUD from HC. Conversely, we tested whether the models trained for classifying MDD vs. HC and AUD vs. HC could distinguish ED patients from healthy controls.

#### Predicting the development of future mental health symptoms

The top 10 reliable variables identified from the classification analyses in the clinical EDs, MDD, and AUD samples were pooled together and used for the prediction analysis in the longitudinal population sample. Data collected at age 14 were used to predict the development of ED symptoms, depressive symptoms, and harmful drinking at age 16/19 years. In addition, we built a second model by adding known risk factors of EDs, including sex, BMI, and pubertal development scale at age 14 to investigate whether they could improve prediction accuracy.

## 3 Results

### 3.1 Characteristics of the samples

In the clinical sample, mean ages ranged from 22.02 to 22.74 years across groups. The AN (N=62) and BN (N=50) groups and their corresponding controls (N=57) were all female. The MDD (N=176) and AUD (N=159) groups and their controls (N=99) involved 75%, 58%, and 59% female participants, respectively (Supplementary Table 1). All the clinical samples were of White ethnicity, except for the control group for AN and BN (81.1% were White). Comorbid mental health conditions were prevalent in the clinical sample: 56.7% of AN and 52.1% of BN patients had current moderate -severe depression, while 3.2% of AN and 14.0% of BN had current severe alcohol problems. 3.3% of MDD and 2.7% of AUD patients screened positive for AN, while 14.6% of MDD and 18.5% of AUD patients screened positive for BN (Supplementary Table 2). In the longitudinal population sample, 1,851 participants (47.4% being female, 88.9% being White) completed the initial assessment at age 14 years and at least one of the two follow-up assessments at ages 16/19 years. From these, we identified developers of ED symptoms (N=221, 59% female) and controls (N=511, 30% female). We also identified 271 developers of depression (62% female) and their 798 controls (46% female), and 522 developers of harmful drinking (39% female) and their 806 controls (55% female). Percentages of missing data are provided in Supplementary Tables 3-5.

### 3.2 Modeling current EDs

Analyses involving all data domains (47 variables) yielded near-perfect classification performance, as measured by area under the receiver operating characteristic curve (AUC-ROC [95% CI]): AN vs HC: 0.97 [0.94-1.00], BN vs. HC: 0.90 [0.83-0.96], AN vs. BN: 0.89 [0.82-0.95]. Expectedly, the high accuracy of classifying AN against the other two groups was dominated by the inclusion of BMI. Rerunning all analyses excluding BMI still yielded a very high AUC-ROC (0.92 [0.86-0.97]) for AN vs. HC classification, indicating that variables other than BMI can still accurately classify AN. For AN vs. BN, the AUC-ROC dropped to 0.75 [0.65-0.83] without BMI but remained significant (*p* < 2.0E-04), while for BN vs. HC, AUC-ROC was almost the same (0.91 [0.85-0.96]), indicating that BMI did not contribute at all to this classification (Figure 1, Supplementary Figure 1). Additional model performance metrics, including area under the precision and recall curve (AUC-PR), sensitivity, specificity, precision, and recall are provided in Supplementary Tables 6-7.

Rerunning analyses with each data domain separately indicated that all domains were accurate classifiers on their own (Figure 1). Personality distinguished all three groups with good accuracies (AUC-ROCs [95% CIs], 0.77-0.89 [0.68-0.95]). Substance use could also distinguish the three groups significantly above chance, albeit with lower accuracies (0.62-0.76 [0.51-0.85]). Psychopathology, environment, and cognition distinguished AN and BN from HC (0.67-0.86 [0.56-0.93]), but not between AN and BN (0.47-0.49 [0.36-0.60], Supplementary Table 6).

**Figure 2:**
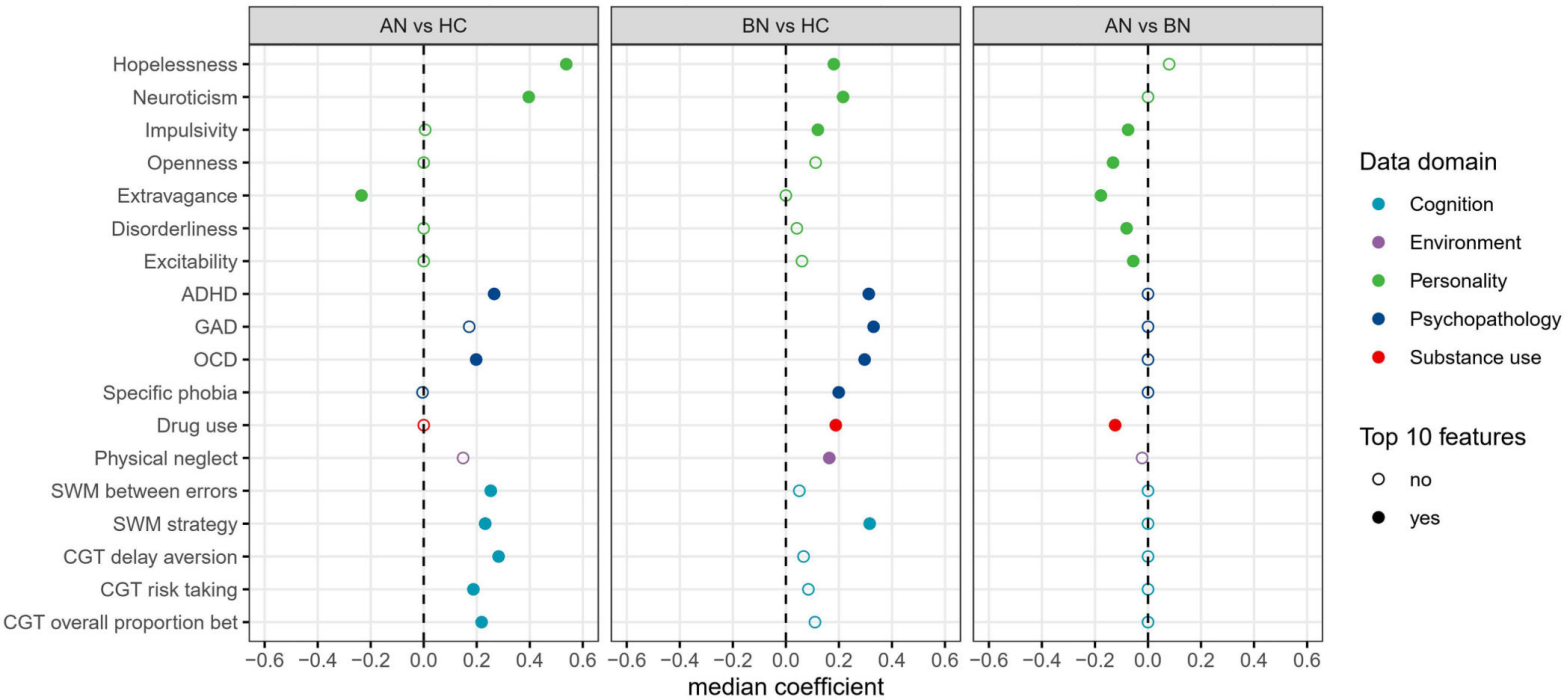
Top 10 reliable features identified from the classification model involving all data domains except BMI. Features are listed if they were among the top 10 reliable features for at least one analysis. Top 10 reliable features are indicated by solid circles. All the features were standardized as z-scores. Feature importance was measured by calculating the median value of the model coefficients across all the cross-validation folds. AN, anorexia nervosa. BN, bulimia nervosa. HC, healthy controls. ADHD, Attention-deficit/hyperactivity disorder. GAD, Generalized anxiety disorder. OCD, Obsessive compulsive disorder. CGT, Cambridge gambling task. SWM, spatial working memory.

We extracted the top 10 reliable features from models including all the data domains except BMI. The features distinguishing both AN and BN from HC included higher neuroticism, hopelessness, symptoms of ADHD and obsessive-compulsive disorder (OCD), and poorer spatial working memory strategies (Figure 2, Supplementary Table 8). The other reliable features distinguishing AN from HC were lower extravagance, executive function and decision making, including more working memory errors, higher delay aversion, risk taking, and the overall proportion of bets. The other reliable contributors to the BN vs. HC classification included symptoms of generalized anxiety disorders (GAD), specific phobia, drug use, and physical neglect. The AN vs. BN analysis identified six reliable features: patients with BN presented higher impulsivity, openness, extravagance, disorderliness, exploratory excitability, and drug use.

### 3.3 Modeling current MDD and AUD

**Figure 3:**
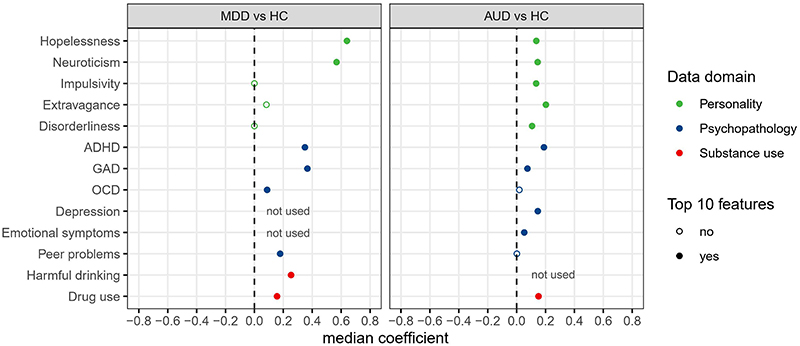
Top 10 reliable features identified from the classification between MDD, AUD, and HC. Features are listed if they were among the top 10 reliable features for at least one analysis. Top 10 reliable features are indicated by solid circles. All the features were standardized as z-scores. AUD, alcohol use disorder. MDD, major depressive disorder. HC, healthy controls. ADHD, Attention-deficit/hyperactivity disorder. GAD, Generalized anxiety disorder. OCD, Obsessive compulsive disorder.

Both MDD (AUC-ROC [95% CI], 0.91 [0.88-0.94]) and AUD (0.80 [0.74-0.85]) could be distinguished from HC with high accuracies (Supplementary Figure 2, Supplementary Table 9). Eight and ten features reliably contributed to the accurate classification of MDD and AUD, respectively (Figure 3, Supplementary Table 10). Five of these reliably classified both disorders from HC, including higher neuroticism, hopelessness, ADHD and GAD symptoms, and drug use. Interestingly, neuroticism, hopelessness, and ADHD symptoms were also among the most contributing features distinguishing both AN and BN from HC. Besides these, reliable features of MDD included OCD symptoms, peer relationship problems, and harmful drinking, while those of AUD included extravagance, disorderliness, impulsivity, depression, and emotional symptoms (Supplementary Table 10).

### 3.4 Transdiagnostic model performance

The models obtained from AN vs. HC and BN vs. HC analyses accurately classified MDD and AUD from HC (AUC-ROCs, 0.75-0.93; *ps* < 2.0E-04 from permutation tests, Supplementary Table 11). The converse was also true: models developed for MDD vs. HC and AUC vs. HC classifications could accurately distinguish AN and BN from HC (AUC-ROCs, 0.83-0.92; *ps* < 2.0E-04 from permutation tests).

### 3.5 Predicting the development of mental health problems

We next tested if the reliable features identified above, when assessed at age 14 in a longitudinal sample, could predict future onset of ED symptoms, depression, and harmful drinking. Data on emotional neglect, physical neglect, and emotional abuse were not available at age 14, and therefore were excluded from analyses. Depressive and emotional symptoms at age 14 were excluded from predicting future depression, and the harmful drinking scale at age 14 was excluded from predicting future harmful drinking. In addition, we excluded three cognitive variables due to excessive missing data: delay aversion, overall proportion of bets, and risk taking (Supplementary Table 5), all from the Cambridge Gambling Task. This resulted in 18, 17, and 16 predictors for ED symptoms, depression, and harmful drinking, respectively.

**Figure 4:**
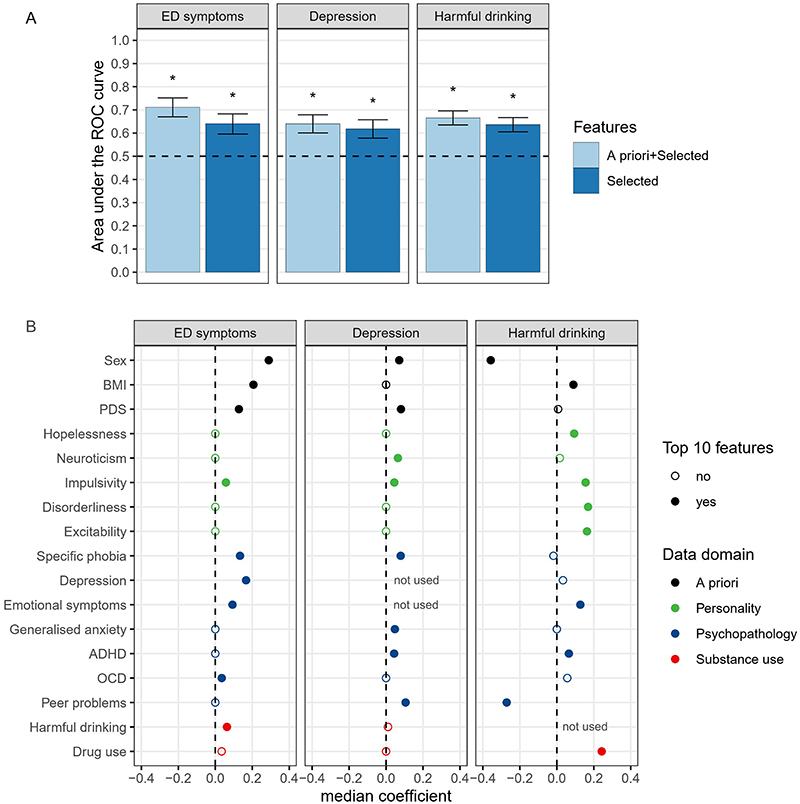
Results of predicting the development of mental health symptoms (A) and top 10 reliable predictors (B). Features are listed if they were among the top 10 reliable features for at least one analysis. Top 10 reliable features are indicated by solid circles. All the features except sex were standardized as z-scores. ROC curve, receiver operating characteristic curve. PDS, pubertal development scale. ADHD, attention-deficit/hyperactivity disorder. OCD, Obsessive compulsive disorder.

The performance was significantly above chance for predicting future ED symptoms (AUC-ROC [95% CI], 0.64 [0.60-0.68]), depressive symptoms (0.62 [0.58-0.66]), and harmful drinking (0.64 [0.61-0.67], Figure 4A, Supplementary Table 12). Adding three known ED risk factors, namely, sex, BMI, and pubertal development improved the model’s performance for predicting future ED symptoms (0.71 [0.67-0,75]), while there was only a minor accuracy increase in predicting depressive symptoms (0.64 [0.60-0.68]) and harmful drinking (0.67 [0.64-0.70], Supplementary Figure 3).

The most reliable predictors for future ED symptoms were being female, having a higher BMI, more advanced pubertal status, symptoms of depression, specific phobia, OCD, emotional symptoms, harmful drinking, and impulsivity. Particularly, higher impulsivity was a common reliable predictor of all three symptoms. Emotional symptoms were not included in the analysis predicting depression, but it was a common reliable predictor of ED symptoms and harmful drinking. Being female, more advanced pubertal status and specific phobia symptoms were shared predictors of ED symptoms and depression. ADHD symptoms were shared predictors of depression and harmful drinking. The other reliable predictors of depression were higher peer relationship problems, neuroticism, and GAD symptoms. On the contrary, lower peer relationship problems were among the top predictors of future harmful drinking, and the other top 10 predictors included drug use, disorderliness, exploratory excitability, hopelessness, and a higher BMI (Figure 4B, Supplementary Table 13).

## 4 Discussion

Our multi-domain analyses combining a wide range of data from clinical and population samples have identified psychosocial profiles predictive of current and future EDs, MDD, and AUD. The classification models built for one disorder were also highly discriminative for the others, indicative of their transdiagnostic potential. Features that distinguished cases from controls also predicted the future onset of ED symptoms, depression, and harmful drinking in a longitudinal adolescent sample. These results demonstrate the value of a multi-domain analysis in predicting both current and future mental illnesses. They also point towards factors that could enhance the effectiveness of early intervention and prevention strategies.

### 4.1 Classification of current AN and BN

While BMI contributed most to the AN classification, the performance of our models was not diminished by excluding BMI. In this respect, our “AN profile” may be a key tool to help eliminate the reliance of healthcare professionals on BMI for AN diagnosis, which has been decried for delaying diagnosis and early intervention (*Position statement on early intervention for eating disorders*, 2019). In fact, DSM-5 now includes a diagnosis of atypical AN where BMI is within or above normal range. Our findings that neuroticism and hopelessness are significantly elevated in EDs corroborated previous findings (Farstad et al., 2016). Hopelessness and depression are significant predictors of suicidal ideation, attempts, and death (Ribeiro et al., 2018). Higher hopelessness may explain the high risk of suicide among patients with EDs (Guillaume et al., 2011). Depression, hopelessness, and suicidal thoughts are common in severe and enduring AN, but in contrast to MDD, antidepressants are not particularly effective in AN. Thus, exploration of novel treatment approaches aimed at improving mood and building hope, e.g., non-invasive neuromodulation, is urgently needed (Gallop et al., 2022).

Features that distinguished AN from BN corroborate the well-established knowledge that substance use is particularly common in BN (Hudson et al., 2007), and that impulsivity (Farstad et al., 2016) and novelty seeking (Bulik et al., 1997; Krug et al., 2009), including disorderliness, extravagance, and exploratory excitability, are shared features of BN and substance use disorders. These features may help improve the stratification of AN and BN, and inform the temperament-based treatment for eating disorders (Kaye et al., 2015).

### 4.2 Classification of current MDD and AUD

The models trained to distinguish EDs from healthy controls were also accurate at classifying AUD and MDD, and vice versa, indicating a high degree of transdiagnostic potential. High neuroticism, hopelessness, and ADHD symptoms characterized all four disorders. The associations between neuroticism, hopelessness, ADHD, and psychiatric disorders have been implicated by previous research investigating each disorder separately. For the first time, we provide evidence for these shared associations in the same study. Genetic associations have been implicated between neuroticism, ADHD, and MDD (Howard et al., 2019), and between ADHD and EDs (Yao et al., 2019). Similar neurobiological alterations in the executive/inhibition and reward systems have been found for ADHD, AUD, and EDs (Casey and Jones, 2010; Seymour et al., 2015; Zhang et al., 2021), suggesting shared mechanisms underlying these conditions.

On the psychopathological level, deficient inhibitory control and delay aversion, both belonging to the impulsivity component of ADHD, are also central features of BN and AUD. People with higher impulsivity are more likely to lose control over consumption of palatable food and alcohol. Delay aversion may lead to binge eating or drinking as a means of seeking immediate pleasure or emotional relief, and purging as a strategy for weight control, rather than adopting problem-solving strategies and healthy lifestyles that take time to yield benefits. The inattention component of ADHD, including difficulties with organizing and planning, may lead to frustration and engagement in disordered eating and binge drinking as coping mechanisms (Herman and Duka, 2019; Kaisari et al., 2017). Alcohol consumption can in turn disrupt impulse control and increase impulsivity, thereby forming a vicious cycle of heavier drinking (Herman and Duka, 2019; Luderer et al., 2021).

Depression typically develops after the onset of ADHD, and previous studies have implicated various mechanisms that may mediate this association. Failures and negative feedback associated with ADHD in academic and social settings may lead to feelings of inadequacy and low self-esteem, which may partly explain the increased risk of depression (Biederman et al., 1998). Other social-environmental mediators include parent-child relationship difficulties, peer relationship problems, and peer victimization (Thapar et al., 2023). Shared risk factors for depression and ADHD may also mediate the pathway, including deficiencies in rewards responsivity, emotion regulation, executive functions and memory (Mayer et al., 2021). An additional pathway from ADHD to depression may involve a third disorder, such as anxiety and disruptive behavior disorders (Thapar et al., 2023).

It should be noted that, as expected, high levels of psychiatric comorbidities were observed in our clinical sample. In particular, more than half of the AN and BN patients had current moderate - severe depression. The shared features identified across diagnoses may therefore reflect these comorbidities. A stringent analysis investigating transdiagnostic potential should ideally include patients without comorbid symptoms, which is difficult to achieve given the high comorbidity and our limited sample size. Future, larger studies are required to clarify this.

On the other hand, the different patterns observed in the psychosocial profiles across disorders highlight the uniqueness and complexity of their shared mechanisms (Munn-Chernoff et al., 2021). Further research is needed to elucidate more detailed mechanisms underlying these mental illnesses.

### 4.3 Predictors of future mental health symptoms

The ability of reliable disease classifiers to predict the later onset of mental health symptoms implies their potential in targeted prevention. Adding well-known ED-related predictors improved prediction accuracies for ED symptoms, highlighting the importance of feature selection in predictive modeling. Consistent with previous research, being female, depressive symptoms, a higher BMI, and pubertal development were among the most potent risk factors for developing ED symptoms (Robinson et al., 2020). Interestingly, pubertal development predicted both future ED and depressive symptoms, which might reflect the impact of being overweight/obese on puberty onset in girls, via the trigger of neuroendocrine processes (Shalitin and Phillip, 2003). A psychosocial process may also play a role: early onset of puberty for young girls confers risk for bullying and harassment (Su et al., 2018), which may in turn contribute to the development of a negative body image, disordered eating behaviors, and depression (Gattario et al., 2020). This calls for early, pre-pubertal interventions in high-risk groups, such as girls with higher BMI, to prevent disease onset (Breton et al., 2022).

Higher impulsivity not only correlated with BN and AUD diagnoses, but also predated the development of ED symptoms and harmful drinking. This suggests that impulsivity may present a common predisposition for these two symptoms to develop. Furthermore, we also identified a temporal relationship indicating that harmful drinking at age 14 increased the risk of future ED symptoms. To date, there have been limited longitudinal studies examining the relationship between EDs and AUD, with emerging evidence indicating that ED symptoms are associated with subsequent alcohol problems (Hirvelä et al., 2023). Our findings, combined with this evidence, suggest a potentially bidirectional relationship between symptoms of EDs and AUD. These findings point towards the need for integrated treatment and prevention strategies that address EDs and AUD simultaneously.

There has been consistent evidence showing that impulsivity is higher in individuals with MDD and is positively associated with depressive symptoms (Fields et al., 2021), but evidence for a longitudinal relationship has been limited (Dussault et al., 2011; Granö et al., 2007). Our results indicate that higher impulsivity is associated with a higher risk of multiple mental health conditions and could be a potential marker in targeted prevention programs. While the reliable predictor in our study was a single measure of impulsivity from the Substance Use Risk Profile Scale (SURPS) (Woicik et al., 2009), it is worth noting that other studies have shown that various facets of impulsivity exhibit differential associations with depressive symptoms (Regan et al., 2019). Further studies are needed to clarify whether specific facets of impulsivity are uniquely associated with particular mental health symptoms.

While being female and having higher peer relationship problems were associated with future depressive symptoms, being male and having lower peer relationship problems elevated risks of future harmful drinking. Although peer relationships consistently correlate with alcohol use in young people, evidence from longitudinal studies has been scarce and inconsistent (Hops et al., 1999; McDonough et al., 2016). Our result may reflect the role of alcohol consumption as a common means of harnessing and developing social connections. During social drinking occasions, factors related to one’s image and reputation among peers are the main drivers of excessive drinking in young people (de Visser et al., 2013), and other factors include coercion and fear of exclusion. Our finding suggests that prevention and early intervention efforts may be enhanced by raising awareness of the social factors contributing to harmful drinking (Brown and Murphy, 2020), in addition to its adverse impact on individuals’ health.

### 4.4 Clinical applications

Clinicians face significant challenges in treating youth psychopathology, as symptoms often manifest and progress differently in young people compared to adults. A lack of confidence, knowledge, and training commonly are cited by primary care practitioners as key barriers to effectively identifying and managing mental health issues in this population (O’Brien et al., 2016). Youth-focused models provide an opportunity to better capture the nuances of psychopathology in young people, enhancing the potential for early detection and targeted intervention. Our findings offer evidence-based insights that are directly relevant to clinical practice. For instance, when assessing overall risk for psychopathology, clinicians may consider impulsivity and sex as shared predictors for EDs, depression and harmful drinking. In addition to these shared risk factors, disorder-specific predictors, such as pubertal development, specific phobia and peer relationship problems, can aid diagnostic differentiation and inform tailored interventions.

Digital tools developed from these youth-focused models represent a promising avenue for addressing gaps in care. These tools can support the early detection of mental health concerns while also providing primary care practitioners with accessible training and educating focused on youth mental health. By integrating such evidence-based approaches and digital tools into clinical practice, primary care providers can be better equipped to address the unique challenges of youth psychopathology, ultimately improving outcomes and well-being for young people.

### 4.5 Limitations

Some limitations should be acknowledged. First, our ED sample involved women only. Also, our study involved predominantly White participants, therefore it remains to be tested how our findings generalize to other ethnic groups. Second, our study did not include some well-known risk factors of EDs such as perfectionism and cognitive inflexibility. Third, while our focus was on the top 10 most reliable features, it should be noted that features beyond the top 10 also made contributions, albeit to a lesser extent. Fourth, while the Elastic Net model offered high interpretability regarding how variables contribute to the outcome, the accuracies for the longitudinal prediction were not adequate for real-world clinical settings. Lastly, the control groups did not report any mental health conditions. Such “clean” control groups provide more interpretable results but tend not to be realistic. Larger and enriched samples and more powerful prediction techniques will be required to achieve better predictability and applicability in future studies.

## 5 Conclusion

Our study demonstrates the capability of machine learning methods to accurately predict mental health diagnoses by leveraging multi-domain psychosocial data. Our findings shed light on crucial aspects influencing mental health outcomes, providing a foundation for targeted prevention and interventions. As we advance our understanding, our work suggests the need for future studies with larger and enriched samples to strengthen the predictive capabilities of machine learning in mental health, fostering a more nuanced and effective approach to diagnosis, intervention, and prevention strategies.

## Supplementary Material

Supplementary

## Data Availability

De-identified data of the IMAGEN, STRATIFY, and ESTRA studies are available to researchers after approval of a proposal by the Executive Committee of these studies (Email: ponscentre@charite.de). The analytical codes are available on the following GitHub repository: https://github.com/crickfan/Predictive-models-in-ED-MDD-AUD

## Highlights

- Psychosocial data and machine-learning models accurately classify psychiatric patients from controls.
- Neuroticism, hopelessness, and ADHD symptoms are transdiagnostic markers.
- BMI information is not needed to for accurate classification of Anorexia Nervosa.
- Development of symptoms at age 16/19y can be predicted based on data from 14y.
