# Supplementary material for: Machine learning models for diagnosis and risk prediction in eating disorders, depression, and alcohol use disorder
Source: J Affect Disord. Author manuscript; Available in PMC 2025 Jun 1. (PMC7617286; doi:10.1016/j.jad.2024.12.053)
Supplement: Supplementary [file EMS202096-supplement-Supplementary.docx]

Machine learning models for diagnosis and risk prediction in eating disorders, depression, and alcohol use disorder

Supplementary information

[Supplementary Methods 2](#_Toc183173650)

[Participants 2](#_Toc183173651)

[Clinical sample 2](#_Toc183173652)

[Population sample 2](#_Toc183173653)

[Exclusion criteria 4](#_Toc183173654)

[Measures 4](#_Toc183173655)

[Cognition 4](#_Toc183173656)

[Environment 4](#_Toc183173657)

[Personality 4](#_Toc183173658)

[Psychopathology 5](#_Toc183173659)

[Substance use 5](#_Toc183173660)

[Body mass index (BMI) 5](#_Toc183173661)

[Pubertal development scale (PDS) 5](#_Toc183173662)

[Confounding variables 6](#_Toc183173663)

[Data analysis 6](#_Toc183173664)

[Partialling out the effect of confounding variables 6](#_Toc183173665)

[Bootstrapping confidence intervals 6](#_Toc183173666)

[Permutation test 6](#_Toc183173667)

[Sample weighting in the prediction models 7](#_Toc183173668)

[Supplementary Tables 8](#_Toc183173669)

[Supplementary Table 1. Characteristics of the samples. 8](#_Toc183173670)

[Supplementary Table 2. Comorbid mental health conditions. 9](#_Toc183173671)

[Supplementary Table 3. Percentage (%) of missing data in each group of the ED sample. 10](#_Toc183173672)

[Supplementary Table 4. Percentage (%) of missing data in each group of the MDD and AUD samples. 12](#_Toc183173673)

[Supplementary Table 5. Percentage (%) of missing data in each group of the population-based IMAGEN sample. 14](#_Toc183173674)

[Supplementary Table 6. Performance of each classification model in the ED sample. 15](#_Toc183173675)

[Supplementary Table 7. Additional performance metrics for the classification models in the ED sample. 16](#_Toc183173676)

[Supplementary Table 8. Top 10 reliable variables identified in ED classification models that involve all variables except BMI. 17](#_Toc183173677)

[Supplementary Table 9. Classification performance for MDD and AUD vs. HC. 18](#_Toc183173678)

[Supplementary Table 10. Top 10 reliable features in the MDD and AUD classifications. 19](#_Toc183173679)

[Supplementary Table 11. Transdiagnostic model performance. 20](#_Toc183173680)

[Supplementary Table 12. Performance of models predicting the development of mental health symptoms in the population sample. 21](#_Toc183173681)

[Supplementary Table 13. Top 10 reliable features in predicting the development of mental health symptoms. 22](#_Toc183173682)

[Supplementary Figures 23](#_Toc183173683)

[**Supplementary Figure 1**.. 23](#_Toc183173684)

[**Supplementary Figure 2**. 24](#_Toc183173685)

[**Supplementary Figure 3**. 25](#_Toc183173686)

[Supplementary References 26](#_Toc183173687)

# Supplementary Methods

## Participants

The three studies involved in our analyses were designed to be both comparable and complementary. IMAGEN is a longitudinal population study of adolescents with the aim of identifying the genetic, psychosocial, and neurobiological basis of developmental psychopathology. The ESTRA and STRATIFY studies, which involve clinical samples, have been established to examine whether markers identified in IMAGEN are still applicable in patients, and vice versa. To ensure consistency, the same standardized study protocols, including the same questionnaires, were employed to assess participants across all three studies. Furthermore, uniform data collection methods and quality control procedures were applied in all three studies.

### Clinical sample

Participants with Anorexia Nervosa (AN) and Bulimia nervosa (BN) were aged 18-25 years and of White ethnicity. They were assessed with the Eating Disorder Diagnostic Scale (EDDS)-DSM5 version ^1^ and met the diagnostic criteria of current Anorexia Nervosa or Bulimia Nervosa.

Participants with Major Depressive Disorder (MDD) and Alcohol Use Disorder (AUD) were aged 18-25 years and of White ethnicity. The MDD group met the criteria of current and acute, moderate - severe depression, assessed by Patient Health Questionnaire-9 (PHQ-9) ^2^, scoring 15 or above. The AUD participants met the criteria of severe alcohol problems, assessed by the Alcohol Use Disorders Identification Test (AUDIT), scoring 15 or above ^3^. Inclusion criteria for the healthy controls were a) PHQ-9 total score < 5; b) AUDIT total score < 5; c) no current/past mental health issues based on self report; d) having no first or second order family members with mental health issues; e) no learning difficulties; f) no regular medication for serious physical health issues; g) no regular recreational drug use.

### Population sample

**Developers of ED symptoms**: Eating disorder symptoms for ages 14, 16 and 19 were obtained from the DAWBA questionnaire ^4^ section P: ‘Dieting, weight and body shape’.

Concerns over shape/weight/eating: Define as providing a positive answer to any of the following items: feeling fat when isn’t (question sp1a); feeling ashamed of eating habits (sp1b); significant interference from eating concerns (sp1d); blaming self a lot for overeating (sp1e); having a fear of gaining weight/getting fat (sp8); feeling terrified of gaining weight/getting fat (sp9); hard to accept weight gain (sp10); thinking of food a lot (sp13); experiencing food cravings like addiction (sp14).

The definitions of binge eating, purging and dieting were the same as in a previous study ^5^.

Binge eating: Defined as answering “Yes” to the following question: “sometimes people lose control over what they eat, and then they eat a very large amount of food in a short time. For example, they may open the fridge and eat as much as they can find – eating and eating until they feel physically ill. This usually happens when people are by themselves. Does this happen to you?” (question 15).

Purging: Defined as answering “Yes” to the question asking if they have ever deliberately made themselves vomit (question 1c), or not answering “Yes” to any of the questions regarding engagement of self-induced vomiting (question 18f) or taking pills or medicines (question 18g) to avoid gaining weight over the last three months. Response options for question 18 include “No”, “I try to but I’m not allowed”, “A little”, or “A lot”.

Dieting: Defined as answering “A lot” to any of the following questions regarding engaging dieting behaviors to avoid gaining weight over the last three months: eating less at meals (question 18a); skipping meals (question 18b) or going without food for long periods, e.g., all day or most of the day (question 18c)”. Response options include “No”, “I try to but I’m not allowed”, “A little”, or “A lot”.

We defined developers of ED symptoms as individuals who did not report any concerns or disordered eating behaviors (binge eating, purging, and dieting) at the initial assessment (age 14) but developed one or more symptoms at age 16 or 19. A control group was defined as individuals who did not report any concerns or disordered eating behaviors across ages 14 to 19.

**Developers of depression**: Depressive symptoms were measured by the DAWBA bands ^6^, which range from 0 to 5, indicating from <0.1% to >70% probability of having a MDD diagnosis ^6^. The developers of depression were defined as those who reported low levels of depression (DAWBA band < 3, indicating <15% risk) at age 14, and higher levels (DAWBA band ≥ 3) at ages 16/19, while the control group included those who reported low levels across the three ages, which followed the definition in a previous study ^5^.

**Developers of harmful drinking**: We defined developers of harmful drinking and controls, by using the AUDIT total score of 8 as cut-off, with a score of 8 or more indicating hazardous or harmful alcohol consumption according to World Health Organization (WHO) guidelines ^3^. The developers were those who reported no drinking or low-risk drinking at age 14, and harmful drinking at ages 16/19. The controls were those who reported no drinking or low-risk drinking across the three ages.

### Exclusion criteria

The ESTRA, STRATIFY and IMAGEN employed similar assessment procedures, and the same exclusion criteria: a) having brain injuries or neurobiological disorders; b) severe hearing or vision difficulties; c) having type I or type II diabetes, or are heavily medicated for serious illness other than for the mental health issues under investigation.; d) being pregnant; e) restricted mobility, including inability to lie flat for 1.5 hours for the MRI scan.

## Measures

### Cognition

The Cognition domain involved measures obtained from the Cambridge Gambling Task (CGT) and Spatial Working Memory (SWM) task in the Cambridge Neuropsychological Test Automated Battery (CANTAB, Cambridge Cognition Ltd, <https://cambridgecognition.com/>). The CGT task provided measures of delay aversion, deliberation time, overall proportion of bets, quality of decision making, risk adjustment and risk taking. The SWM task provided measures of between errors and strategy. We also involved verbal comprehension and perceptual reasoning indices obtained from the Wechsler Adult Intelligence Scale-Fourth Edition (Pearson plc, London, UK).

### Environment

The environmental data domain involved childhood trauma experiences assessed by the Childhood Trauma Questionnaire (CTQ) ^7^, and experience of bullying measured by the revised Olweus Bully/Victim Questionnaire ^8^. The CTQ included measures of emotional abuse, emotional neglect, physical abuse, physical neglect, and sexual abuse. The sexual abuse scale was not available in our dataset due to technical errors. CTQ was not administrated in IMAGEN at age 14.

We involved five items in the revised Olweus Bully/Victim Questionnaire, including being bullied/victimized at school/college/work, being bullied by a family member, being isolated, or being emotionally or physically victimized.

### Personality

The personality domain included the Revised NEO Five-Factor Inventory (NEO-FFI) ^9^, Substance Use Risk Profile Scale (SURPS) ^10^, and Temperament and Character Inventory – Revised (TCI-R, only the novelty seek facet was administrated) ^11^. The NEO-PI-R included subscales of openness, conscientiousness, extraversion, agreeableness, and neuroticism. The SURPS included subscales of anxiety sensitivity, hopelessness, impulsivity, and sensation-seeking. The novelty-seeking facet of the TCI-R included subscales of exploratory excitability, impulsiveness, extravagance, and disorderliness.

### Psychopathology

Five subscales of the Strengths and Difficulties Questionnaire ^12^ were involved, including emotional symptoms, conduct problems, hyperactivity/inattention, peer relationship problems, and prosocial behaviors.

The Development and Well-Being Assessment (DAWBA) is a set of questionnaires, interviews, and rating techniques designed to generate psychiatric diagnoses. Computer-generated DAWBA bands ^6^ were used to measure the severity of psychopathological symptoms. DAWBA bands comprised up to 6 levels (from 0 to 5) and indicated the probability of having a disorder (from <0.1% to >70% probability of DSM-IV based diagnoses). The following psychopathological symptoms were included in our analysis: depression, generalized anxiety, obsessive-compulsive disorder, panic disorder, post-traumatic stress disorder, specific phobia, and social phobia.

### Substance use

The total score of the Alcohol Use Disorders Identification Test (AUDIT) ^13^ was used to measure harmful drinking. Drug use frequencies in the past year were obtained from the ESPAD questionnaire ^14^, summing up the frequencies of using the following drugs: amphetamines (speed, uppers, crystal meth) or desoxyn, anabolic steroids, cocaine, crack, GHB or liquid ecstasy, inhalants (glue, aerosols etc.), marijuana (grass, pot) or hashish (hash, hash oil), heroin, ketamine, LSD (acid), ecstasy (MDMA), mushrooms, narcotics (e.g. opium, morphine, codeine), tranquillizers, or sedatives (without a doctor’s prescription).

### Body mass index (BMI)

BMI (kg/m^2^) was derived from height and weight measurements. BMI at age 14 years in the IMAGEN cohort was transformed to age- and sex-adjusted z-scores based on the Centre for Disease Control and Prevention Growth Chart ^15^.

### Pubertal development scale (PDS)

The Pubertal Development Scale ^16^ was used to assess pubertal development at age 14 years in the IMAGEN cohort. It is a self-report measure of pubertal development, including growth spurts in height, body hair growth, and skin changes for both boys and girls; breast development and menarche for girls only; and voice change and facial hair growth for boys only. The answer to each item was coded in a four-level ordinal scale. The mean value across the five items was used as an overall measure of pubertal development for each individual.

### Confounding variables

In the classification analysis involving the ED samples, age was considered a confounding variable, as all the participants were female and recruited from London. In the analysis of MDD and AUD samples, sex was a confounding variable in addition to age and study site. This was due to unbalanced sex between cases and controls. In the analysis of the population IMAGEN sample, confounding variables included age and study site, while sex was used as a predictor.

## Data analysis

### Partialling out the effect of confounding variables

The effects of confounding variables were partialled out from the training and testing data separately by using linear regression, following the procedure recommended by Snoek et al. (2019) ^17^. For each feature in the training data, a linear regression model was fitted with the confounding variables being the predictors. Residuals from this model were obtained and used for model training. This linear regression model was directly applied to the testing data (without model refitting) to obtain residuals of each feature. This approach ensured that no information from the testing data was utilized in model training.

### Bootstrapping confidence intervals

Confidence intervals of the performance metrics (AUC-ROC and AUC-PR) were obtained by using bootstrapping. For each repeat of the CV, the model’s output was resampled with repetition. Based on the resampled values the performance metrics were obtained. This procedure was repeated 2000 times for each repeat of the CV, forming a bootstrap distribution. The lower and upper bounds of the CI were derived from the 2.5% and 97.5% percentile of the bootstrapping distribution and averaged across the 10 repetitions.

### Permutation test

P values for the model’s performance were obtained from permutation tests. We randomly shuffled the group membership of samples before submitting the data to the same CV procedure described above, and derived performance metrics. This procedure was repeated 5000 times to derive null distributions of AUC-ROC and AUC-PR. To calculate the P-value, we counted how many values in the null distribution exceeded the actual performance and divided this count by the total number of permutations.

### Sample weighting in the prediction models

In building the prediction models using the longitudinal IMAGEN data, the training and testing procedures were the same as those used for the clinical sample, except that sample weights were provided for the model training to deal with group size imbalances between the developers and controls. The weight of a sample was inversely proportional to the group size, thus assigning higher weights to the developers than the controls.

# Supplementary Tables

Supplementary Table 1. Characteristics of the samples.

| Sample type | | Group | Group size | Female (%) | BMI Mean (SD) | Age Mean (SD) |
| --- | --- | --- | --- | --- | --- | --- |
| Clinical sample | EDs | AN | 62 | 100% | 16.56* (1.88) | 22.02 (2.19) |
|  |  | BN | 50 | 100% | 23.41* (4.30) | 22.17 (2.29) |
|  |  | HC for EDs | 57 | 100% | 25.24 (5.46) | 22.63 (0.77) |
|  | MDD and AUD | MDD | 176 | 75% | 24.46 (6.08) | 22.73 (2.19) |
|  |  | AUD | 159 | 58% | 23.41 (4.29) | 22.74 (2.20) |
|  |  | HC for MDD and AUD | 99 | 59% | 23.56 (4.14) | 22.43 (1.45) |
| Population sample | Development of ED symptoms | Future ED symptoms | 221 | 59% | 20.70* (3.27) | 14.37 (0.52) |
|  |  | Controls for ED symptoms | 511 | 30% | 19.62 (2.74) | 14.45 (0.51) |
|  | Development of depression | Future depressive symptoms | 271 | 62% | 20.74 (3.26) | 14.43 (0.52) |
|  |  | Controls for depression | 798 | 46% | 20.48 (3.27) | 14.44 (0.52) |
|  | Development of harmful drinking | Future harmful drinking | 522 | 39% | 20.96* (3.32) | 14.44 (0.51) |
|  |  | Controls for harmful drinking | 806 | 55% | 20.57 (3.40) | 14.43 (0.52) |

Footnote: An asterisk (*) indicates a significant difference (*p*<0.05) in the mean value compared with the control group, by using a two-sample t-test. Age and BMI in the IMAGEN sample are from the baseline assessment at age 14 years. The developers’ groups in the population sample were not mutually exclusive. ED, eating disorder.AN, anorexia nervosa. BN, bulimia nervosa. MDD, major depressive disorder. AUD, alcohol use disorder. HC, healthy control.

Supplementary Table 2. Comorbid mental health conditions.

| Comorbidities in the AN and BN groups | | | | |
| --- | --- | --- | --- | --- |
| Patient group | Group size | PHQ9 and AUDIT data available (N) | Moderate - severe depression  (PHQ-9 total score>=15) | Severe alcohol problems  (AUDIT total score>=15) |
| AN | 62 | 60, 62 | 34 (56.7%) | 2 (3.2%) |
| BN | 50 | 48, 50 | 25 (52.1%) | 7 (14.0%) |
| Comorbidities in the MDD and AUD groups | | | | |
| Patient group | Group size | EDDS data available (N) | AN  (EDDS screen) | BN  (EDDS screen) |
| MDD | 176 | 151 | 5 (3.3%) | 22 (14.6%) |
| AUD | 159 | 146 | 4 (2.7%) | 27 (18.5%) |

Footnote: Diagnoses of AN and BN were assessed using the Eating Disorder Diagnosis Scale (EDDS)^18^ and DSM-5 based criteria.

Supplementary Table 3. Percentage (%) of missing data in each group of the ED sample.

| Variable | Assessment name | AN | BN | ED controls |
| --- | --- | --- | --- | --- |
| BMI | - | 6.45 | 8 | 0 |
| Hopelessness | SURPS | 0 | 0 | 0 |
| Anxiety sensitivity | SURPS | 0 | 0 | 0 |
| Impulsivity | SURPS | 0 | 0 | 0 |
| Sensation seeking | SURPS | 0 | 0 | 0 |
| Neuroticism | NEO-FFI | 0 | 0 | 0 |
| Extraversion | NEO-FFI | 0 | 0 | 0 |
| Openness | NEO-FFI | 0 | 0 | 0 |
| Agreeableness | NEO-FFI | 0 | 0 | 0 |
| Conscientiousness | NEO-FFI | 0 | 0 | 0 |
| Exploratory excitability | TCI-R | 0 | 0 | 0 |
| Impulsivity | TCI-R | 0 | 0 | 0 |
| Extravagance | TCI-R | 0 | 0 | 0 |
| Disorderliness | TCI-R | 0 | 0 | 0 |
| Perceptual Reasoning | WAISE-IV | 12.9 | 12 | 3.51 |
| Verbal Comprehension | WAISE-IV | 12.9 | 12 | 3.51 |
| CGT-Delay aversion | CANTAB | 3.23 | 4 | 0 |
| CGT-Deliberation time | CANTAB | 3.23 | 4 | 0 |
| CGT-Overall proportion bet | CANTAB | 3.23 | 4 | 0 |
| CGT-Quality of decision making | CANTAB | 3.23 | 4 | 0 |
| CGT-Risk adjustment | CANTAB | 3.23 | 4 | 0 |
| CGT-Risk taking | CANTAB | 3.23 | 4 | 0 |
| SWM-Between errors | CANTAB | 3.23 | 4 | 0 |
| SWM-Strategy | CANTAB | 3.23 | 4 | 0 |
| Hyperactivity/ inattention | SDQ | 3.23 | 10 | 1.75 |
| Conduct problems | SDQ | 3.23 | 10 | 1.75 |
| Emotional symptoms | SDQ | 3.23 | 10 | 1.75 |
| Peer relationship problems | SDQ | 3.23 | 10 | 1.75 |
| Prosocial behaviors | SDQ | 3.23 | 10 | 1.75 |
| Depression | DAWBA | 6.45 | 20 | 1.75 |
| Generalized anxiety | DAWBA | 6.45 | 20 | 1.75 |
| Obsessive compulsive disorder | DAWBA | 6.45 | 20 | 1.75 |
| Panic disorder | DAWBA | 6.45 | 16 | 1.75 |
| Post-traumatic stress disorder | DAWBA | 6.45 | 16 | 1.75 |
| Social phobia | DAWBA | 6.45 | 16 | 1.75 |
| Specific phobia | DAWBA | 6.45 | 14 | 1.75 |
| Harmful drinking | AUDIT | 0 | 0 | 0 |
| Drug use | ESPAD | 0 | 0 | 0 |
| Emotional abuse | CTQ | 3.23 | 4 | 1.75 |
| Physical abuse | CTQ | 3.23 | 4 | 1.75 |
| Emotional neglect | CTQ | 6.45 | 10 | 1.75 |
| Physical neglect | CTQ | 3.23 | 8 | 1.75 |
| Bullied at school/ college/ work | Revised Olweus Bully/ Victim | 0 | 0 | 0 |
| Emotional bullying by a peer | Revised Olweus Bully/ Victim | 0 | 0 | 0 |
| Bullied with social exclusion by a peer | Revised Olweus Bully/ Victim | 0 | 0 | 0 |
| Physical bullying by a peer | Revised Olweus Bully/ Victim | 0 | 0 | 0 |
| Bullied by family | Revised Olweus Bully/ Victim | 0 | 0 | 0 |
| Age in years | - | 9.68 | 8 | 0 |

Footnote: Age is a confounding variable. CGT, Cambridge gambling task. SWM, spatial working memory.

Supplementary Table 4. Percentage (%) of missing data in each group of the MDD and AUD samples.

| Variable | Assessment name | MDD | AUD | Controls |
| --- | --- | --- | --- | --- |
| BMI | - | 10.23 | 6.92 | 3.03 |
| Hopelessness | SURPS | 0 | 0 | 1.01 |
| Anxiety sensitivity | SURPS | 0 | 0 | 1.01 |
| Impulsivity | SURPS | 0 | 0 | 1.01 |
| Sensation seeking | SURPS | 0 | 0 | 1.01 |
| Neuroticism | NEO-FFI | 0 | 0 | 1.01 |
| Extraversion | NEO-FFI | 0 | 0 | 1.01 |
| Openness | NEO-FFI | 0 | 0 | 1.01 |
| Agreeableness | NEO-FFI | 0 | 0 | 1.01 |
| Conscientiousness | NEO-FFI | 0 | 0 | 1.01 |
| Exploratory excitability | TCI-R | 0 | 0 | 1.01 |
| Impulsivity | TCI-R | 0 | 0 | 1.01 |
| Extravagance | TCI-R | 0 | 0 | 1.01 |
| Disorderliness | TCI-R | 0 | 0 | 1.01 |
| Perceptual Reasoning* | WAISE-IV | 14.20 | 18.87 | 48.48 |
| Verbal Comprehension* | WAISE-IV | 14.77 | 18.87 | 48.48 |
| CGT-Delay aversion* | CANTAB | 17.05 | 20.75 | 3.03 |
| CGT-Deliberation time* | CANTAB | 16.48 | 20.75 | 3.03 |
| CGT-Overall proportion bet* | CANTAB | 16.48 | 20.75 | 3.03 |
| CGT-Quality of decision making* | CANTAB | 16.48 | 20.75 | 3.03 |
| CGT-Risk adjustment* | CANTAB | 16.48 | 20.75 | 3.03 |
| CGT-Risk taking* | CANTAB | 16.48 | 20.75 | 3.03 |
| SWM-Between errors* | CANTAB | 16.48 | 20.75 | 3.03 |
| SWM-Strategy* | CANTAB | 16.48 | 20.75 | 3.03 |
| Hyperactivity/ inattention | SDQ | 3.98 | 2.52 | 3.03 |
| Conduct problems | SDQ | 3.98 | 2.52 | 3.03 |
| Emotional symptoms | SDQ | Not used | 2.52 | 3.03 |
| Peer relationship problems | SDQ | 3.98 | 2.52 | 3.03 |
| Prosocial behaviors | SDQ | 3.98 | 2.52 | 3.03 |
| Depression | DAWBA | Not used | 3.14 | 3.03 |
| Generalized anxiety | DAWBA | 5.68 | 3.14 | 3.03 |
| Obsessive compulsive disorder | DAWBA | 5.68 | 3.14 | 3.03 |
| Panic disorder | DAWBA | 5.68 | 3.14 | 3.03 |
| Post-traumatic stress disorder | DAWBA | 5.68 | 3.14 | 3.03 |
| Social phobia | DAWBA | 5.68 | 3.14 | 3.03 |
| Specific phobia | DAWBA | 5.11 | 3.14 | 3.03 |
| Harmful drinking | DAWBA | 0 | Not used | 0 |
| Drug Use | ESPAD | 0 | 0 | 0 |
| Emotional abuse* | CTQ | 26.14 | 8.81 | 32.32 |
| Physical abuse* | CTQ | 25.57 | 8.81 | 31.31 |
| Emotional neglect* | CTQ | 26.70 | 8.81 | 32.32 |
| Physical neglect* | CTQ | 27.27 | 8.18 | 30.30 |
| Bullied at school/college/work | Revised Olweus Bully/ Victim | 0 | 0 | 0 |
| Emotional bullying by a peer | Revised Olweus Bully/ Victim | 0 | 0 | 0 |
| Bullied with social exclusion by a peer | Revised Olweus Bully/ Victim | 0 | 0 | 0 |
| Physical bullying by a peer | Revised Olweus Bully/ Victim | 0 | 0 | 0 |
| Bullied by family | Revised Olweus Bully/ Victim | 0 | 0 | 0 |
| Sex | - | 0 | 0 | 0 |
| Age in years | - | 0.57 | 0 | 0 |
| Study site | - | 0 | 0 | 0 |

Footnote: Variables labelled with asterisks (*) were excluded from classification analysis due to excessive and imbalanced missing data across the groups. Sex, age, and study sites are confounding variables. Variables closely related to the diagnosis are labelled “not used” and excluded from analysis. CGT, Cambridge gambling task. SWM, spatial working memory.

Supplementary Table 5. Percentage (%) of missing data in each group of the population-based IMAGEN sample.

| Variable | ED symptoms developers | ED controls | Depression developers | Depression controls | Harmful drinking developers | Harmful drinking controls |
| --- | --- | --- | --- | --- | --- | --- |
| sex | 0 | 0 | 0 | 0 | 0 | 0 |
| BMI | 10.41 | 6.26 | 9 | 5.26 | 4.8 | 5.09 |
| PDS | 9.95 | 8.81 | 12.84 | 9.15 | 11.07 | 7.07 |
| Hopelessness | 0 | 1.37 | 0.96 | 1.25 | 1.11 | 0.62 |
| Impulsivity | 0 | 1.37 | 0.96 | 1.25 | 1.11 | 0.62 |
| Neuroticism | 0 | 0.59 | 0.38 | 1 | 0.74 | 0.62 |
| Openness | 0 | 0.59 | 0.57 | 1 | 0.74 | 0.62 |
| Exploratory excitability | 0 | 0.98 | 0.19 | 0.63 | 0.74 | 0.37 |
| Extravagance | 0 | 0.98 | 0.19 | 0.63 | 0.74 | 0.37 |
| Disorderliness | 0 | 0.98 | 0.19 | 0.63 | 0.74 | 0.37 |
| CGT-delay aversion* | 19.46 | 13.31 | 19.16 | 14.91 | 17.34 | 14.76 |
| CGT-overall proportion bet* | 19.46 | 13.11 | 18.97 | 14.79 | 17.34 | 14.76 |
| CGT-risk taking* | 19.46 | 13.11 | 18.97 | 14.79 | 17.34 | 14.76 |
| SWM-between errors | 6.33 | 2.15 | 5.17 | 3.13 | 3.69 | 3.6 |
| SWM-strategy | 6.33 | 2.15 | 5.17 | 3.13 | 3.69 | 3.6 |
| Hyperactivity/ inattention | 0 | 0 | 0.38 | 0 | 0 | 0 |
| Emotional symptoms | 0 | 0 | Not used | Not used | 0 | 0 |
| Peer relationship problems | 0 | 0 | 0.38 | 0 | 0 | 0 |
| Depression | 0 | 0 | Not used | Not used | 0 | 0 |
| Generalized anxiety | 0 | 0 | 0.38 | 0 | 0 | 0 |
| Obsessive compulsive disorder | 0 | 0 | 0.38 | 0 | 0 | 0 |
| Specific phobia | 0 | 0 | 0.38 | 0 | 0 | 0 |
| Drug use | 0 | 0 | 0 | 0.13 | 0 | 0 |
| Harmful drinking | 0.9 | 0.2 | 0 | 0.63 | Not used | Not used |
| Age in years | 3.17 | 0.98 | 2.68 | 1.25 | 0.74 | 1.49 |
| Study site | 0 | 0 | 0 | 0 | 0 | 0 |

Footnote: Variables labelled with asterisks (*) were excluded from analysis due to excessive and imbalanced missingness across the developer and control groups. Age and study sites are confounding variables. Variables closely related to the outcome are labelled “not used” and excluded from analysis. CGT, Cambridge gambling task. SWM, spatial working memory.

Supplementary Table 6. Performance of each classification model in the ED sample.

| Analysis | Domain | AUC-ROC | | | AUC-PR | | | |
| --- | --- | --- | --- | --- | --- | --- | --- | --- |
|  |  | Mean | Bootstrap 95% CI | P value | Mean | Bootstrap 95% CI | Chance level | P value |
| AN vs. HC | All | 0.97 | 0.94-1.00 | <2.0E-04* | 0.97 | 0.93-1.00 | 0.52 | <2.0E-04* |
|  | BMI | 0.95 | 0.92-0.98 | <2.0E-04* | 0.96 | 0.93-0.99 | 0.52 | <2.0E-04* |
|  | Without BMI | 0.92 | 0.86-0.97 | <2.0E-04* | 0.92 | 0.85-0.98 | 0.52 | <2.0E-04* |
|  | Cognition | 0.74 | 0.65-0.83 | <2.0E-04* | 0.76 | 0.65-0.86 | 0.52 | <2.0E-04* |
|  | Environment | 0.67 | 0.56-0.76 | 0.0028* | 0.71 | 0.58-0.82 | 0.52 | 0.0016* |
|  | Personality | 0.89 | 0.82-0.95 | <2.0E-04* | 0.89 | 0.80-0.96 | 0.52 | <2.0E-04* |
|  | Psychopathology | 0.83 | 0.75-0.90 | <2.0E-04* | 0.87 | 0.78-0.93 | 0.52 | <2.0E-04* |
|  | Substance use | 0.62 | 0.51-0.72 | 0.017* | 0.65 | 0.53-0.78 | 0.52 | 0.014* |
| BN vs. HC | All | 0.90 | 0.83-0.96 | <2.0E-04* | 0.89 | 0.80-0.97 | 0.47 | <2.0E-04* |
|  | BMI | 0.58 | 0.47-0.69 | 0.086 | 0.54 | 0.42-0.69 | 0.47 | 0.12 |
|  | Without BMI | 0.91 | 0.85-0.96 | <2.0E-04* | 0.91 | 0.83-0.97 | 0.47 | <2.0E-04* |
|  | Cognition | 0.78 | 0.69-0.86 | <2.0E-04* | 0.77 | 0.65-0.87 | 0.47 | <2.0E-04* |
|  | Environment | 0.68 | 0.57-0.78 | 0.0030* | 0.68 | 0.55-0.81 | 0.47 | 0.0016* |
|  | Personality | 0.88 | 0.80-0.94 | <2.0E-04* | 0.87 | 0.78-0.95 | 0.47 | <2.0E-04* |
|  | Psychopathology | 0.86 | 0.78-0.93 | <2.0E-04* | 0.88 | 0.81-0.95 | 0.47 | <2.0E-04* |
|  | Substance use | 0.76 | 0.66-0.85 | <2.0E-04* | 0.78 | 0.66-0.88 | 0.47 | <2.0E-04* |
| AN vs. BN | All | 0.89 | 0.82-0.95 | <2.0E-04* | 0.91 | 0.83-0.97 | 0.55 | <2.0E-04* |
|  | BMI | 0.93 | 0.89-0.97 | <2.0E-04* | 0.95 | 0.90-0.99 | 0.55 | <2.0E-04* |
|  | Without BMI | 0.75 | 0.65-0.83 | <2.0E-04* | 0.76 | 0.64-0.88 | 0.55 | 0.0010* |
|  | Cognition | 0.48 | 0.38-0.59 | 0.56 | 0.55 | 0.44-0.68 | 0.55 | 0.63 |
|  | Environment | 0.47 | 0.36-0.58 | 0.63 | 0.54 | 0.43-0.67 | 0.55 | 0.68 |
|  | Personality | 0.77 | 0.68-0.86 | <2.0E-04* | 0.80 | 0.70-0.90 | 0.55 | <2.0E-04* |
|  | Psychopathology | 0.49 | 0.38-0.60 | 0.51 | 0.59 | 0.47-0.72 | 0.55 | 0.34 |
|  | Substance use | 0.72 | 0.62-0.82 | <2.0E-04* | 0.75 | 0.63-0.86 | 0.55 | 6.0E-04* |

Footnote: P values were derived from permutation tests with 5000 permutes. Asterisks (*) indicate that the P value survived FDR correction for multiple comparisons (false discovery rate, FDR<0.05 for the 24 tests = 8 domains × 3 analyses). The chance-level of AUC-ROC is 0.5 for all the analyses. AUC-ROC, area under the receiver operating characteristic curve; AUC-PR, area under the precision-recall curve; CI, confidence interval.

Supplementary Table 7. Additional performance metrics for the classification models in the ED sample.

| Analysis | Domain | ROC curve | | PR curve | |
| --- | --- | --- | --- | --- | --- |
|  |  | Sensitivity | Specificity | Precision | Recall |
| AN vs. HC | all | 0.95 | 0.93 | 0.93 | 0.95 |
|  | BMI | 0.88 | 0.90 | 0.90 | 0.89 |
|  | Without BMI | 0.88 | 0.89 | 0.89 | 0.90 |
|  | Cognition | 0.73 | 0.69 | 0.67 | 0.82 |
|  | Environment | 0.51 | 0.84 | 0.57 | 0.90 |
|  | Personality | 0.82 | 0.89 | 0.87 | 0.84 |
|  | Psychopathology | 0.69 | 0.90 | 0.81 | 0.75 |
|  | Substance use | 0.55 | 0.72 | 0.53 | 0.99 |
| BN vs. HC | all | 0.85 | 0.89 | 0.87 | 0.85 |
|  | BMI | 0.64 | 0.59 | 0.48 | 0.99 |
|  | Without BMI | 0.86 | 0.90 | 0.87 | 0.87 |
|  | Cognition | 0.81 | 0.63 | 0.63 | 0.89 |
|  | Environment | 0.59 | 0.78 | 0.58 | 0.82 |
|  | Personality | 0.88 | 0.80 | 0.79 | 0.89 |
|  | Psychopathology | 0.73 | 0.90 | 0.80 | 0.79 |
|  | Substance use | 0.57 | 0.90 | 0.65 | 0.79 |
| AN vs. BN | all | 0.85 | 0.87 | 0.87 | 0.89 |
|  | BMI | 0.88 | 0.87 | 0.88 | 0.91 |
|  | Without BMI | 0.69 | 0.76 | 0.69 | 0.86 |
|  | Cognition | 0.70 | 0.37 | 0.55 | 0.99 |
|  | Environment | 0.64 | 0.44 | 0.56 | 1.00 |
|  | Personality | 0.73 | 0.78 | 0.73 | 0.84 |
|  | Psychopathology | 0.41 | 0.69 | 0.56 | 1.00 |
|  | Substance use | 0.88 | 0.53 | 0.69 | 0.91 |

Footnote: Sensitivity and specificity were derived from a point on the receiver operating characteristic (ROC) curve where their sum was the highest. Precision and recall values were derived a point from the precision-recall (PR) curve where their harmonic mean (i.e., F1-score) was the highest.

Supplementary Table 8. Top 10 reliable variables identified in ED classification models that involve all variables except BMI.

| Analysis | Variable | Selection ratio (%) | Median standardized coefficient |
| --- | --- | --- | --- |
| AN vs. HC | Hopelessness | 100 | 0.54 |
|  | Neuroticism | 100 | 0.40 |
|  | CGT-Delay aversion | 100 | 0.28 |
|  | Hyperactivity/ inattention | 100 | 0.27 |
|  | SWM-Between errors | 100 | 0.25 |
|  | Extravagance | 100 | -0.23 |
|  | SWM-Strategy | 100 | 0.23 |
|  | CGT-Overall proportion bet | 100 | 0.22 |
|  | Obsessive compulsive disorder | 100 | 0.20 |
|  | CGT-Risk taking | 100 | 0.19 |
| BN vs. HC | Generalized anxiety disorder | 100 | 0.33 |
|  | SWM-Strategy | 100 | 0.32 |
|  | Hyperactivity/ inattention | 100 | 0.31 |
|  | Obsessive compulsive disorder | 100 | 0.30 |
|  | Neuroticism | 100 | 0.22 |
|  | Specific phobia | 99 | 0.20 |
|  | Drug Use | 99 | 0.19 |
|  | Hopelessness | 100 | 0.18 |
|  | Physical neglect | 100 | 0.16 |
|  | SURPS-Impulsivity | 100 | 0.12 |
| AN vs. BN | Extravagance | 100 | -0.18 |
|  | Openness | 99 | -0.13 |
|  | Drug Use | 99 | -0.12 |
|  | Disorderliness | 98 | -0.08 |
|  | SURPS-Impulsivity | 98 | -0.08 |
|  | Exploratory excitability | 97 | -0.06 |

Footnote: This table presents variables with a selection ratio ≥ 0.9 in the classification analyses. If more than 10 variables meet this criterion, the top 10 are listed. Median standardized coefficients were obtained across the models from all the cross-validation folds. SURPS, Substance Use Risk Profile Scale.

Supplementary Table 9. Classification performance for MDD and AUD vs. HC.

|  |  | MDD vs. HC | AUD vs. HC |
| --- | --- | --- | --- |
| ROC curve | Mean AUC | 0.91 | 0.80 |
|  | Bootstrap 95% CI | 0.88-0.94 | 0.74-0.85 |
|  | Chance level | 0.50 | 0.50 |
|  | P value | <2.0E-04 | <2.0E-04 |
|  | Sensitivity | 0.82 | 0.60 |
|  | Specificity | 0.92 | 0.92 |
| PR curve | Mean AUC | 0.95 | 0.88 |
|  | Bootstrap 95% CI | 0.93-0.98 | 0.83-0.92 |
|  | Chance level | 0.64 | 0.62 |
|  | P value | <2.0E-04 | <2.0E-04 |
|  | Precision | 0.91 | 0.70 |
|  | Recall | 0.86 | 0.92 |

P values were derived from permutation tests with 5000 permutes. ROC curve, receiver operating characteristic curve; PR curve, precision-recall curve; AUC, area under the curve; CI, confidence interval.

Supplementary Table 10. Top 10 reliable features in the MDD and AUD classifications.

| Analysis | Variable | Selection ratio (%) | Median standardized coefficient | Reliability of feature in the ED classification models |
| --- | --- | --- | --- | --- |
| MDD vs. HC | Hopelessness | 100 | 0.64 | AN, BN>HC |
|  | Neuroticism | 100 | 0.57 | AN, BN>HC |
|  | Generalized anxiety disorder | 100 | 0.37 | BN>HC |
|  | Hyperactivity/ inattention | 100 | 0.35 | AN, BN>HC |
|  | Peer relationship problems | 98 | 0.18 | - |
|  | Harmful drinking | 94 | 0.25 | - |
|  | Obsessive compulsive disorder | 94 | 0.09 | AN, BN>HC |
|  | Drug Use | 90 | 0.16 | BN>AN, HC |
| AUD vs. HC | Extravagance | 100 | 0.20 | BN>AN; HC>AN |
|  | Hyperactivity/ inattention | 100 | 0.19 | AN, BN>HC |
|  | Drug Use | 100 | 0.15 | BN>AN, HC |
|  | Depression | 100 | 0.15 | - |
|  | Neuroticism | 100 | 0.15 | AN, BN>HC |
|  | Hopelessness | 100 | 0.14 | AN, BN>HC |
|  | SURPS-Impulsivity | 100 | 0.14 | BN>AN, HC |
|  | Disorderliness | 100 | 0.11 | BN>AN |
|  | Generalized anxiety disorder | 100 | 0.07 | BN>HC |
|  | Emotional symptoms | 98 | 0.05 | - |

Footnote: This table presents variables with a selection ratio ≥ 0.9 in the classification analyses. If more than 10 variables meet this criterion, the top 10 are listed. Median standardized coefficients were obtained across the models from all the cross-validation folds. The last column lists whether a feature is among the top 10 reliable features for the ED classification models in Supplementary 7. SURPS, Substance Use Risk Profile Scale.

Supplementary Table 11. Transdiagnostic model performance.

| Source data | Test data | AUC-ROC | P value | AUC-PR | P value |
| --- | --- | --- | --- | --- | --- |
| AN vs. HC | MDD vs. HC | 0.93 | <2.0E-04 | 0.97 | <2.0E-04 |
|  | AUD vs. HC | 0.75 | <2.0E-04 | 0.86 | <2.0E-04 |
| BN vs. HC | MDD vs. HC | 0.92 | <2.0E-04 | 0.96 | <2.0E-04 |
|  | AUD vs. HC | 0.79 | <2.0E-04 | 0.89 | <2.0E-04 |
| MDD vs. HC | AN vs. HC | 0.92 | <2.0E-04 | 0.93 | <2.0E-04 |
|  | BN vs. HC | 0.90 | <2.0E-04 | 0.90 | <2.0E-04 |
| AUD vs. HC | AN vs. HC | 0.83 | <2.0E-04 | 0.86 | <2.0E-04 |
|  | BN vs. HC | 0.92 | <2.0E-04 | 0.93 | <2.0E-04 |

Footnote: Classification models were trained on the source data and model performance was obtained from the test data. BMI was excluded from analysis. P values were derived from permutation tests with 5000 permutes. The healthy controls (HC) for the AN and BN groups did not overlap with the controls for MDD and AUD. AUC-ROC, area under the receiver operating characteristic curve; AUC-PR, area under the precision-recall curve.

Supplementary Table 12. Performance of models predicting the development of mental health symptoms in the population sample.

|  | Analysis | ED symptoms | | Depression | | Harmful drinking | |
| --- | --- | --- | --- | --- | --- | --- | --- |
|  | Domain | A priori + Selected | Selected | A priori + Selected | Selected | A priori + Selected | Selected |
| ROC curve | Mean AUC | 0.71 | 0.64 | 0.64 | 0.62 | 0.67 | 0.64 |
|  | Bootstrap 95% CI | 0.67-0.75 | 0.60-0.68 | 0.60-0.68 | 0.58-0.66 | 0.64-0.70 | 0.61-0.67 |
|  | Chance level | 0.50 | 0.50 | 0.50 | 0.50 | 0.50 | 0.50 |
|  | P value | <2.0E-04 | <2.0E-04 | <2.0E-04 | <2.0E-04 | <2.0E-04 | <2.0E-04 |
|  | Sensitivity | 0.70 | 0.65 | 0.57 | 0.56 | 0.59 | 0.63 |
|  | Specificity | 0.65 | 0.58 | 0.66 | 0.65 | 0.68 | 0.59 |
| PR curve | Mean AUC | 0.51 | 0.43 | 0.38 | 0.36 | 0.56 | 0.53 |
|  | Bootstrap 95% CI | 0.44-0.58 | 0.37-0.50 | 0.33-0.44 | 0.31-0.42 | 0.51-0.60 | 0.48-0.57 |
|  | Chance level | 0.30 | 0.30 | 0.25 | 0.25 | 0.39 | 0.39 |
|  | P value | <2.0E-04 | <2.0E-04 | <2.0E-04 | <2.0E-04 | <2.0E-04 | <2.0E-04 |
|  | Precision | 0.45 | 0.38 | 0.33 | 0.32 | 0.46 | 0.45 |
|  | Recall | 0.74 | 0.76 | 0.70 | 0.69 | 0.86 | 0.81 |

P values were derived from permutation tests with 5000 permutes. “A priori” variables included sex, BMI, and pubertal development scale. “Selected” variables included the top 10 reliable variables identified from the classification analyses in the clinical samples, which are listed in Supplementary tables 7 and 10. ROC curve, receiver operating characteristic curve; PR curve, precision-recall curve; AUC, area under the curve; CI, confidence interval.

Supplementary Table 13. Top 10 reliable features in predicting the development of mental health symptoms.

| Group of developers | Variable | Selection ratio | Median standardized coefficient |
| --- | --- | --- | --- |
| ED symptoms | Sex | 100 | 0.29 |
|  | BMI | 100 | 0.21 |
|  | Depression | 100 | 0.17 |
|  | Specific phobia | 100 | 0.13 |
|  | Pubertal development scale | 100 | 0.13 |
|  | Emotional symptoms | 100 | 0.09 |
|  | Harmful drinking | 98 | 0.06 |
|  | SURPS-Impulsivity | 97 | 0.06 |
|  | Obsessive compulsive disorder | 94 | 0.03 |
| Depression | Peer relationship problems | 100 | 0.11 |
|  | Pubertal development scale | 100 | 0.08 |
|  | Specific phobia | 100 | 0.08 |
|  | Sex | 100 | 0.07 |
|  | Neuroticism | 100 | 0.06 |
|  | Generalized anxiety disorder | 100 | 0.05 |
|  | SURPS-Impulsivity | 100 | 0.04 |
|  | Hyperactivity/ inattention | 100 | 0.04 |
| Harmful drinking | Sex | 100 | -0.36 |
|  | Peer relationship problems | 100 | -0.27 |
|  | Drug use | 100 | 0.24 |
|  | Disorderliness | 100 | 0.17 |
|  | Exploratory excitability | 100 | 0.16 |
|  | SURPS-Impulsivity | 100 | 0.16 |
|  | Emotional symptoms | 100 | 0.13 |
|  | Hopelessness | 100 | 0.09 |
|  | BMI | 100 | 0.09 |
|  | Hyperactivity/ inattention | 100 | 0.07 |

Footnote: This table presents variables with a selection ratio ≥ 0.9 in the predictive models built on the IMAGEN sample. If more than 10 variables meet this criterion, the top 10 are listed. Median standardized coefficients were obtained across the models from all the cross-validation folds. SURPS, Substance Use Risk Profile Scale.

# Supplementary Figures


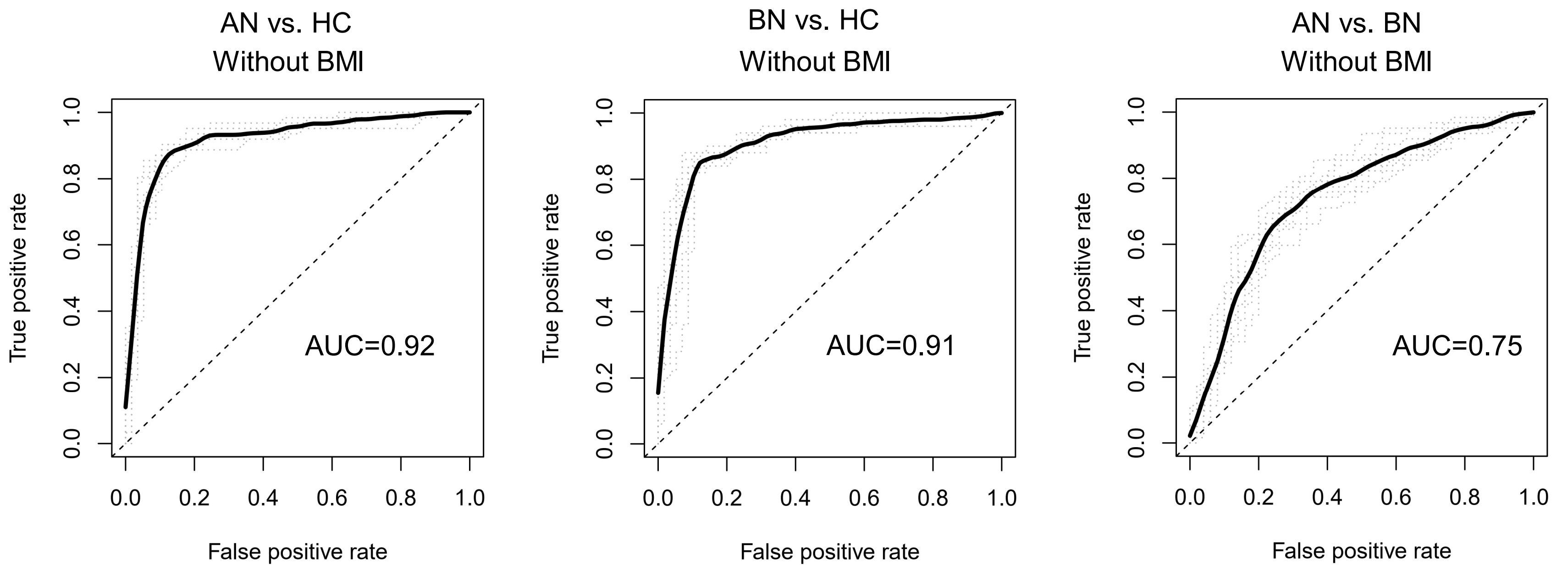


**Supplementary Figure 1**. The receiver operating characteristic curve for the classification models for AN and BN, involving all the features except BMI. Solid lines represent the average curve across the 10 repetitions of cross-validation. Dashed lines represent the curve for each repetition of cross-validation. AUC, area under the curve. The ROC curves were plotted with the ROCR package ([https://CRAN.R-project.org/package=ROCR](https://cran.r-project.org/package=ROCR)).


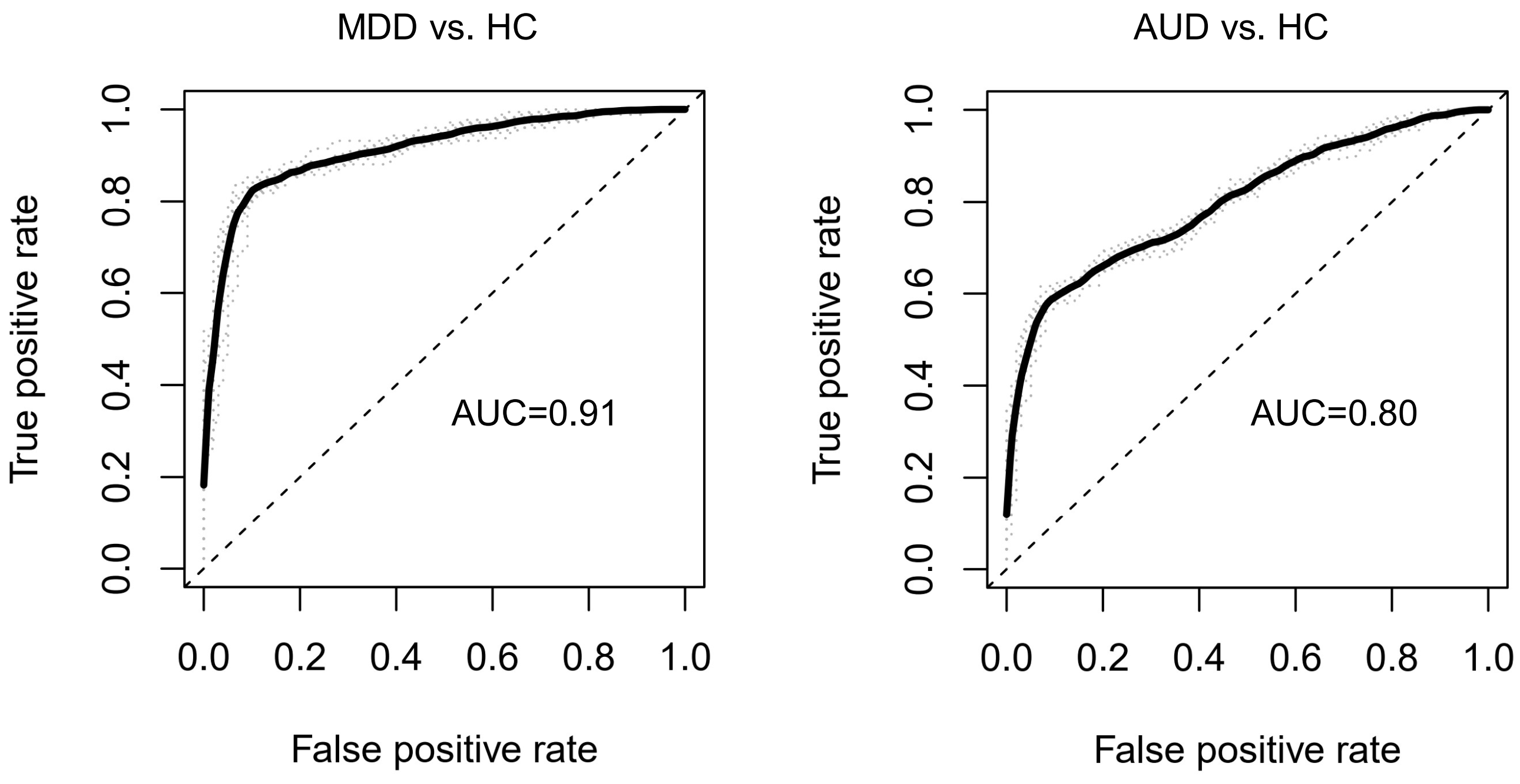


**Supplementary Figure 2**. The receiver operating characteristic curve for the classification models for MDD and AUD. Solid lines represent the average curve across the 10 repetitions of cross-validation. Dashed lines represent the curve for each repetition of cross-validation. AUC, area under the curve


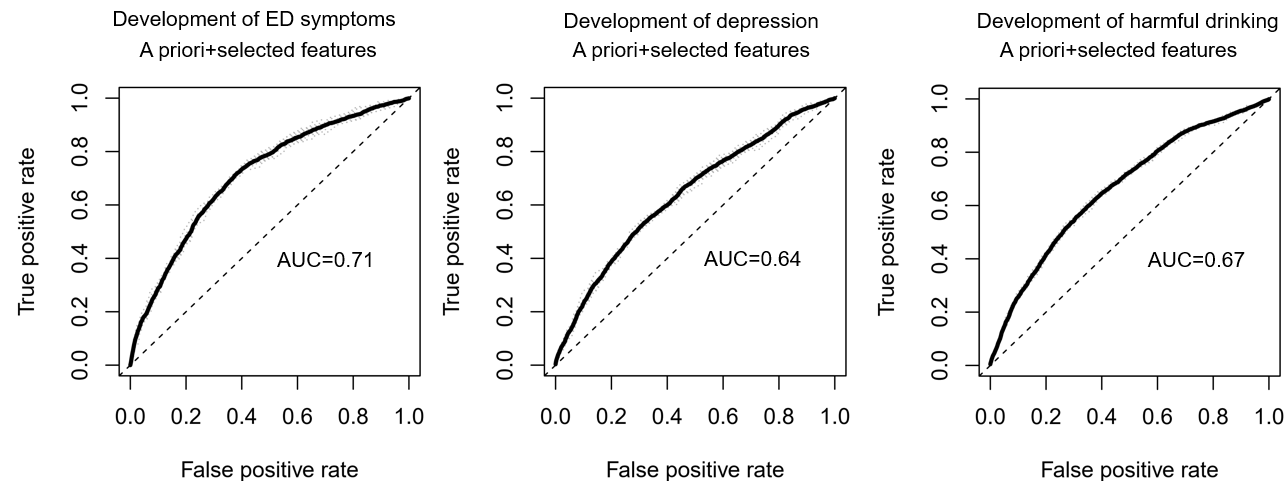


**Supplementary Figure 3**. The receiver operating characteristic curve for the predictive models in the longitudinal IMAGEN sample. Solid line represents the average curve across the 10 repetitions of cross-validation. Dashed lines represent the curve for each repetition of cross-validation. AUC, area under the curve

# Supplementary References

1 Bohon C, Stice E. Eating Disorder Diagnostic Scale. In *Encyclopedia of Feeding and Eating Disorders* (ed T Wade ): 254–7. Springer, 2017.

2 Kroenke K, Spitzer RL, Williams JB. The PHQ-9: validity of a brief depression severity measure. *J Gen Intern Med* 2001; **16**: 606–13.

3 Babor TF, Higgins-Biddle JC, Saunders JB, Monteiro MG. AUDIT : the Alcohol Use Disorders Identification Test : guidelines for use in primary health care (second edition). 2001. (https://www.who.int/publications/i/item/WHO-MSD-MSB-01.6a).

4 Goodman R, Ford T, Richards H, Gatward R, Meltzer H. The Development and Well-Being Assessment: description and initial validation of an integrated assessment of child and adolescent psychopathology. *J Child Psychol Psychiatry* 2000; **41**: 645–55.

5 Zhang Z, Robinson L, Jia T, Quinlan EB, Tay N, Chu C, *et al.* Development of Disordered Eating Behaviors and Comorbid Depressive Symptoms in Adolescence: Neural and Psychopathological Predictors. *Biol Psychiatry* 2021; **90**: 853–62.

6 Goodman A, Heiervang E, Collishaw S, Goodman R. The ‘DAWBA bands’ as an ordered-categorical measure of child mental health: description and validation in British and Norwegian samples. *Soc Psychiatry Psychiatr Epidemiol* 2011; **46**: 521–32.

7 Bernstein DP, Fink L. *Childhood trauma questionnaire: a retrospective self-report*. Psychological Corporation, 1998.

8 Olweus D. *The revised Olweus Bully/Victim Questionnaire for students*. Research Center for Health Promotion (HEMIL Center), University of Bergen, 1996.

9 Costa PT, McCrae RR. *The NEO-PI/NEO-FFI manual supplement*. Psychological Assessment Resources, 1989.

10 Woicik PA, Stewart SH, Pihl RO, Conrod PJ. The Substance Use Risk Profile Scale: a scale measuring traits linked to reinforcement-specific substance use profiles. *Addict Behav* 2009; **34**: 1042–55.

11 Cloninger CR, Przybeck TR, Svrakic DM. The Temperament and Character Inventory-Revised. Center for Psychobiology of Personality, Washington University, 1999.

12 Goodman R. The Strengths and Difficulties Questionnaire: a research note. *J Child Psychol Psychiatry* 1997; **38**: 581–6.

13 Saunders JB, Aasland OG, Babor TF, de la Fuente JR, Grant M. Development of the Alcohol Use Disorders Identification Test (AUDIT): WHO Collaborative Project on Early Detection of Persons with Harmful Alcohol Consumption--II. *Addiction* 1993; **88**: 791–804.

14 Hibell B, Anderson B, Bjarnason T, Kokkevi A, Morgan M, Narusk A. The 1995 ESPAD Report. Alcohol and Other Drug Use Among Students in 26 European Countries. The Swedish Council for Information on Alcohol and Other Drugs, 1997.

15 Flegal KM, Cole TJ. Construction of LMS parameters for the Centers for Disease Control and Prevention 2000 growth charts. *National health statistics reports* 2013; **63**: 1–3.

16 Petersen AC, Crockett L, Richards M, Boxer A. A self-report measure of pubertal status: Reliability, validity, and initial norms. *J Youth Adolesc* 1988; **17**: 117–33.

17 Snoek L, Miletić S, Scholte HS. How to control for confounds in decoding analyses of neuroimaging data. *NeuroImage* 2019; **184**: 741–60.

18 Stice E, Telch CF, Rizvi SL. Development and validation of the Eating Disorder Diagnostic Scale: a brief self-report measure of anorexia, bulimia, and binge-eating disorder. *Psychol Assess* 2000; **12**: 123–31.
